# Transcriptome Analysis Reveals Vimentin-Induced Disruption of Cell–Cell Associations Augments Breast Cancer Cell Migration

**DOI:** 10.3390/cells11244035

**Published:** 2022-12-13

**Authors:** Saima Usman, Ahmad Jamal, Antesar Bushaala, Naushin H. Waseem, Hebah Al-Dehlawi, William Andrew Yeudall, Muy-Teck Teh, Hemanth Tummala, Ahmad Waseem

**Affiliations:** 1 Centre for Oral Immunobiology and Regenerative Medicine, Institute of Dentistry, Barts and The London School of Medicine and Dentistry, Queen Mary University of London, Newark Street, London E1 2AT, UK; 2 St Bartholomew’s Hospital, Barts NHS Trust, King George V Building, 5th Floor, London EC1A 7BE, UK; 3 Department of Oral Diagnostic Sciences, Division of Oral Pathology and Medicine, Faculty of Dentistry, King Abdul Aziz University, Jeddah 21589, Saudi Arabia; 4 Department of Oral Biology and Diagnostic Sciences, The Dental College of Georgia, Augusta University, Augusta, GA 30912, USA; 5 Centre for Genomics and Child Health, Blizard Institute, Barts and The London School of Medicine and Dentistry, Queen Mary University of London, Turner Street, London E1 2AT, UK

**Keywords:** cancer cell migration, cytokeratins, cell proliferation, RNA-Seq, cell–cell junctions

## Abstract

In advanced metastatic cancers with reduced patient survival and poor prognosis, expression of vimentin, a type III intermediate filament protein is frequently observed. Vimentin appears to suppress epithelial characteristics and augments cell migration but the molecular basis for these changes is not well understood. Here, we have ectopically expressed vimentin in MCF-7 and investigated its genomic and functional implications. Vimentin changed the cell shape by decreasing major axis, major axis angle and increased cell migration, without affecting proliferation. Vimentin downregulated major keratin genes *KRT8*, *KRT18* and *KRT19*. Transcriptome-coupled GO and KEGG analyses revealed that vimentin-affected genes were linked to either cell–cell/cell-ECM or cell cycle/proliferation specific pathways. Using shRNA mediated knockdown of vimentin in two cell types; MCF-7FV (ectopically expressing) and MDA-MB-231 (endogenously expressing), we identified a vimentin-specific signature consisting of 13 protein encoding genes (*CDH5*, *AXL*, *PTPRM*, *TGFBI*, *CDH10*, *NES*, *E2F1*, *FOXM1*, *CDC45*, *FSD1*, *BCL2*, *KIF26A* and *WISP2*) and two long non-coding RNAs, *LINC00052* and *C15ORF9-AS1*. CDH5, an endothelial cadherin, which mediates cell–cell junctions, was the most downregulated protein encoding gene. Interestingly, downregulation of CDH5 by shRNA significantly increased cell migration confirming our RNA-Seq data. Furthermore, presence of vimentin altered the lamin expression in MCF-7. Collectively, we demonstrate, for the first time, that vimentin in breast cancer cells could change nuclear architecture by affecting lamin expression, which downregulates genes maintaining cell–cell junctions resulting in increased cell migration.

## 1. Introduction

Intermediate filaments (IFs) are a large family of structural polypeptides which are expressed only in metazoans. In humans, there are about 70 different IF genes that are divided into 6 types (type I to VI) based on physiochemical characteristics, sequence homology and tissue-specific expression [1]. Keratins are heteropolymers of type I and type II IFs and are expressed primarily in epithelia [2]. Vimentin is a type III IF polypeptide which forms homopolymers and is normally expressed in mesenchymal tissues. During oncogenic transformation, cells begin to express vimentin which is a reliable marker of epithelial mesenchymal transition (EMT)-induced metastasis with poor prognosis [3,4,5]. In addition, there is loss of keratins, especially K8/18, in epithelial cancers [6]. In metastatic cells, keratin and vimentin may be co-expressed, and the two filament systems have been shown to interact with each other [7], although they do not co-polymerise [8]. 

Assortment of organelles at specific places within a cell is vital for their optimal functions, and vimentin in consort with actin and Rac1 has been shown to play a key role in providing stable position to a number of cell organelles [9] including the nucleus [10], Golgi complex [11], melanosomes [12] and mitochondria [13] to maintain cellular homeostasis. Vimentin has been shown to inhibit osteoblast differentiation [14], and it can bear various kind of stresses such as electrophilic, heat shock and oxidative by disassembling into an oligomeric state [15]. It protects differentiating stem cells from stress by forming a cage around stress granules [16]. Vimentin delivers extracellular cues intracellularly by ECM remodelling via two mechanisms: first, stabilising type 1 collagen mRNA, thereby synthesising more collagen 1 [17], and by modulating attachment of the cell to ECM [18]. It is involved in assembly of focal adhesions (FA)—short squiggles of vimentin localise at nascent FA while filamentous vimentin is associated with mature FAs [19]. At FAs, vimentin has been shown to bind with a number of proteins including plectin, filamin A and fimbrin [20,21,22] to regulate the dynamics of FA and control cell migration. In addition, vimentin provides mechanical viscoelastic support, intracellular and extracellular redistribution of tractional and shear forces, cortical stiffness, maintenance of cell polarity via orchestrating microtubules and moreover influencing multiple signalling pathways active during cell migration [23]. 

The nucleoskeleton is linked to the cytoskeleton via Linkers of the Nucleoskeleton to the Cytoskeleton (LINC) complex, which is responsible for diverse functions including nuclear integrity, positioning, mechanics and genome organization and stability [24]. The nuclear lamins (A, C, B1 and B2), are present on the inner surface of the nuclear envelope and associate with vimentin as part of the LINC complex [25]. These lamin-vimentin associations can maintain nucleocellular morphology and mechanics [26].

Several reports have shown that motile and invasive cells express higher levels of vimentin [27,28] and its absence attenuates migration of cancer cells [29,30]. Hendrix and co-workers first showed that vimentin expression in MCF-7 cells (which lack vimentin) increases migration and its downregulation in MDA-MB-231 cells (which show robust expression of vimentin) suppressed cell migration [31]. Several mechanisms have been proposed as the basis for vimentin-mediated enhanced migration, although the mechanistic details are unclear and poorly explored. In this study, we performed transcriptome profiling using RNA-Seq analysis of MCF-7 ectopically expressing vimentin to identify the molecular basis of this complex process. We show that cytonuclear transduction induced by vimentin generates signalling causing reduction in the expression of proteins at cell–cell junctions, thereby increasing cell migration. 

## 2. Materials and Methods

### 2.1. Cell Culture and Cell Lines

The human cell lines used in this study, their origins and sources along with references where these cells lines were either made or first used are listed in Table S1 [32,33,34,35,36,37,38,39,40,41,42,43,44,45,46,47,48,49,50,51,52,53]. These were cultured in either Dulbecco’s Modified Eagle Medium (DMEM), containing 10% (*v*/*v*) foetal calf serum (FCS), 50 units/mL penicillin and 50 μg/mL streptomycin (complete medium) or in Rheinwald Green Modified (RM+) growth medium consisting of a 3:1 ratio of DMEM:F12 (Nutrient mixture Ham’s 12), with 10% FCS, penicillin/streptomycin, insulin to a final concentration of 5 μg/mL, liothyronine 2 × 10^−11^ M, transferrin 5 μg/mL, cholera toxin at 8.4 ng/mL, hydrocortisone 0.4 μg/mL, epidermal growth factor 10 ng/mL, adenine 24 μg/mL. Cells were maintained in a humidified incubator in an atmosphere of 5% CO_2_ + 95% air at 37 °C.

### 2.2. Antibodies

Primary antibodies used were: mouse monoclonal to vimentin (abcam, Cambridge, UK, Cat # ab 8069; 1:2000 dilution); rabbit polyclonal to GAPDH (abcam, Cambridge, UK, Cat # ab9485; 1:2000 dilution), lamin B2 (Thermofisher, Oxford, UK, Cat # PA5-29121, 1:500), CDH5 (abcam, Cambridge, UK, Cat # ab33168; 1:1000); rabbit monoclonal to laminA/C (Thermofisher, Oxford, UK, Cat # MA5-35284; 1:1000); lamin B1 (Thermofisher, Oxford, UK, Cat # 702972; 1:500). Secondary antibodies used were: goat anti-mouse IgG peroxidase conjugated (GE healthcare, Lisburn, UK, Cat # NA931V, 1:1000 dilution), donkey anti-Rabbit IgG peroxidase conjugated (GE healthcare, Lisburn, UK, Cat # NA934; 1:1000 dilution). 

### 2.3. RNA Extraction and cDNA Synthesis

Cells were cultured in six well plate format in triplicates and lysed in 500 µL Dynabeads mRNA lysis buffer when about 70% confluent. Invitrogen Dynabeads mRNA Direct Kit (Cat # 610-12; Thermofisher Scientific, Oxford, UK) was used for mRNA extractions from the cells. A total of 50 ng mRNA from each cell line was used to prepare the cDNA with a mixture of oligo-dT and random hexamers using the qPCRBIO cDNA Synthesis Kit (Cat # PB30.11-10, PCR Biosystems, London, UK) in triplicates according to the manufacturer’s instructions. For total RNA extraction, Qiagen RNeasy Kit (Cat # 74104) was used according to the manufacturer’s protocol. The reverse transcription reaction was carried out at 42 °C for 30 min, heat inactivated at 85 °C for 5 min and cooled to 4 °C for 5 min. cDNA was diluted 1:20 with RNase/DNase-free water and stored at −80 °C until used. 

### 2.4. Quantitative PCR

Quantitative PCR (qPCR) was performed with a Light Cycler 480 qPCR system (Roche, Burgess Hill, West Sussex, UK) using Light Cycler 480 SYBR Green I Master reaction mix (PCR Biosystems, London, UK) as described previously [54]. Forward and reverse primers for the genes studied here are listed in Table S2. Results were normalized with two reference genes: *YAP1* and *POLR2A* [54]. 

### 2.5. Protein Extraction and Western Blotting

Cells were cultured in 6 well plates in triplicates. When 70–80% confluent, cells were washed twice with PBS and lysed using 250 μL/well Laemmli lysis buffer (2% sodium dodecyl sulphate (SDS), 20% glycerol, 125 mM Tris-HCl, pH 6.8). SDS gel electrophoresis and Western blotting were performed as described previously [55]. ECL prime detection reagent (Cat # PRN2232, GE Healthcare, Lisburn, UK) and ChemiDoc (BioRad, Watford, UK) were used to visualise protein bands. Quantification of the band intensities was performed using *Image J* software [56].

### 2.6. Plasmid Constructs, cDNA Cloning, Retrovirus Production and Spinfection

The original plasmid pLPC-N MYC, a puromycin selectable vector, and a gift from Dr Titia de Lange (Addgene plasmid # 12540), was digested with HindIII and XhoI and ligated with double stranded DNA containing 10 different restriction sites (F: 5′AGCTGGAATTCATCGATACCGGTGGGCCCAGATCTGTATACAAGCTTGGATCCGTCGACTTCGAAAATC3′; R: 5′TCGAGATTTTCGAAGTCGACGGATCCAAGCTTGTATACAGATCTGGGCCCACCGGTATCGATGAATTCC3′). To make pLPChygro, an AvrII site at the 5′ end (F: 5′CTAGGCGCCGGCCGGATCAGCCTAGGATGACCGAGTACAAGCCCACGGTG3′; R: 5′CACCGTGGGCTTGTACTCGGTCATCCTAGGCTGATCCGGCCGGCGCCTAG3′) and a BsmI site at the 3′ end (F: GACCCGCAAGCCCGGTGCCTGAGAATGCCCCCACGACCCGCAGCGCCCGACC; R: GGTCGGGCGCTGCGGGTCGTGGGGGCATTCTCAGGCACCGGGCTTGCGGGTC) of the puromycin cassette was created by site-directed mutagenesis using the method described elsewhere [57] except that Q5 DNA polymerase (NEB, Hitchin, UK) was used for PCR and Taq DNA ligase (NEB, Hitchin, UK) was added to seal the newly synthesised DNA. The puromycin cassette was then swapped with hygromycin cassette. 

The full-length human vimentin cDNA was sub-cloned into pLPChygro as described previously [55]. The constructs were named pLPChygro-FV (full-length vimentin) and pLPChygro-CV (control vector)**.** For vimentin knockdown, cloning of VIM-shRNA along with non-target control (NTC) in *pSiren-Retro-Q* has been described previously [30]. The primers used to make 4 different shRNA constructs for CDH5 and for NTC are listed in Table S3. The forward and reverse primers were annealed, phosphorylated and ligated into *pSuper.retro.puro* previously digested with BglII and XhoI. All clones were sequenced before use. For packaging puromycin constructs, a one-step amphotropic retrovirus production was employed using Phoenix A cells [58]. For hygromycin constructs, a two-step method involving ecotropic retrovirus in the first step followed by an amphotropic virus production in the second step was used [59]. 

For retroviral transduction, 50,000 MCF-7 cells were seeded in T25 culture flasks in complete medium. The viral supernatant and culture cells were treated with 5 µg/mL hexadimethrine bromide (polybrene) for 20–30 min. The medium was then replaced with the polybrene/virus mixture and the flasks were spun at 1000 rpm at 32 °C for 1h and the cells were selected with hygromycin as described previously [59]. MCF-7FV and MDA-MB-231 cells were transduced with shVIM and shNTC retroviruses as described above and selected with 1 μg/mL puromycin. All transduced cell lines used in this study are listed in Table 1.

### 2.7. Analysis of Cell Parameters

MCF-7FV and CV were fixed with 4% (*w*/*v*) paraformaldehyde in PBS (pH 7.4) for 20 min and their bright field images were acquired using a Nikon Eclipse TE2000-S microscope. To analyse different cell morphological features, 10,000 cells were seeded in 96 well plates in triplicate. After they had attached, cells were washed with PBS, fixed with 4% (*w*/*v*) paraformaldehyde in PBS and permeabilised with 0.1% (*v*/*v*) Triton-X in PBS for 20 min. Cells were stained with CellMask^TM^ Deep Red (Cat # H32721; Invitrogen, Paisley, UK) (1 µL in 200 mL) and DAPI (1 μg/mL), incubated for 2 h and then washed with PBS 3× times. Cell morphology was analysed using INCA 2200 IN Cell Analyzer GE Widefield System. More than 2000 cells were counted from each cell line using GE IN Carta software (INCarta Cytiva, Marlborough, MA, USA).

### 2.8. Colony Assay

MCF-7FV and CV were seeded at a density of 500 cells/well in 6 well plates. Cells were allowed to grow for 2 weeks. Subsequently they were washed with PBS, fixed with ice cold methanol for 30 min followed by staining with 0.1% (*w*/*v*) crystal violet dye in 25% (*v*/*v*) methanol for 2 h at RT. The plates were rinsed to remove excess stain, dried and scanned. The area covered by colonies were measured using *ImageJ* software [56].

### 2.9. CyQUANT^TM^ Cell Proliferation and Cell Adhesion Assays

Cell proliferation and adhesion assays were performed using Molecular Probes™ CyQUANT™ Assay kit (Cat # C7026), according to the manufacturer’s protocol. Cells were plated in a 12 well plate and when 70% confluent, trypsinised and counted using a haemocytometer. For the generation of standard curve, a cell suspension was made at the density of 10^6^ cells/mL in complete DMEM without phenol red in 1.5 mL Eppendorf tubes. This cell suspension was centrifuged at 1500rpm in a microcentrifuge, supernatant was discarded, and the pellet was stored at −70 °C. The cell pellet was thawed from −70 °C to RT and 1 mL of CyQUANT GR dye/lysis buffer was added to each pellet, and the lysate was resuspended by vortexing. A dilution series from 50 to 50,000 cells was created in 96 well culture plate with CyQUANT GR dye/lysis buffer, in final volumes of 200 μL per well in triplicates. Control of 200 μL CyQUANT GR dye/lysis buffer/well without cells was also prepared in triplicates as controls. The samples were incubated in dark for 5 min at RT. The fluorescence of each sample was measured with ClarioStar microplate reader with 480 nm (±10 nm) excitation and 520 nm (±15 nm) emission filters. Standard calibration curve was generated by plotting fluorescence values versus cell number. For proliferation assay at different time points, 5000 cells in triplicates were platted in 96 well culture plates for 24 and 48 h in complete DMEM without phenol red. Different plates were used for each time point and controls without cells were also included in each plate. The plates were incubated at 37 °C for a time sufficient to allow the cells to attach and grow. Every other day, 100 µL medium was removed from each well and replaced with fresh medium. At the desired time, medium was removed, and the cells were washed with PBS. Cells were frozen in the 96 well plate and stored at −70 °C until samples were assayed. When ready to quantitate the samples, plates were thawed at room temperature, then 200 µL of the CyQUANT^®^ GR dye/cell-lysis buffer was added to each sample well and assayed as described above. For each time point cell number was determined using the standard curve generated earlier. 

For adhesion assay, two 96 well plates were used. A total of 20,000 cells from each cell line in triplicates were plated to allow the cells to attach. After 8 h when most of the cells had attached, one plate was centrifuged at 1500× *g* for 15 min and supernatant was removed. This was used for the total cell count. The other 96 well plate was washed with PBS twice to remove the unattached cells and after final wash these plates were frozen at −80 °C. Later, the cells were counted according to manufacturer’s instructions for CyQUANT kit (Cat # C7026) as mentioned above for CyQUANT cell proliferation assay. 

### 2.10. MTT Assay

MTT stock (12 mM) solution was prepared in PBS by dissolving 50 mg of 3-(4,5-Dimethylthiazol-2-yl)-2,5-Diphenyltetrazolium Bromide (MTT) powder in 10 mL PBS and filtered. For standard curve, MCF-7 cells were seeded at the density of 250, 500, 1250, 2500, 6250, 12,500, 25,000 and 100,000 cells in triplicates in 200 µL complete medium without phenol red in a 96 well plate that was earlier coated with 10 µg/mL collagen for 1 h at 37 °C. The cells were allowed to attach for 8 h after which the medium was removed from the cells and 100 µL of complete medium without phenol red containing 10 µL of MTT solution was added into each well. Cells were incubated at 37 °C for 3 h until purple color was observed. Soluble MTT containing medium was carefully removed from the cells and 100 µL DMSO was added in each well to dissolve purple formazan crystals and incubated for 10 min. Absorbance was read at 570 nm in a microplate reader. A calibration curve was plotted using absorbance versus cell number. For measuring the metabolic activity in MCF-7 cells expressing FV and CV, the cells were grown in 96 well plates in sextuplets in complete medium without phenol red at 37 °C in the tissue culture incubator for 12 h, 24 h, 36 h, 48 h and 60 h. Separate plates were used for different time points and the wells having no cells were used as control. After incubation for the required time, medium was removed, and plates were assayed as described above. Viable cell number was determined by converting the OD_570_ into cell number using the calibration curve [60].

### 2.11. Wound Healing Assay

Cell migration was evaluated using Oris^TM^ Cell Migration Assay (Cat # CMAU101, Platypus Technologies, Oxford, UK) according to the manufacturer’s protocol. Between 60,000–70,000 cells/100 µL/well in 96 well plates were seeded around culture inserts and allowed to attach overnight. Next day, the cells were treated with 10 µg/mL mitomycin C (Sigma-Merck, Gillingham, Dorset, UK). After 4 h, the culture inserts were removed, and cells were allowed to migrate. Images were taken at 4× objective by Nikon Eclipse TE2000-S microscope at different time intervals. Area covered by the cells at each time was measured by *ImageJ* software [56].

### 2.12. Chemotactic Migration

Cells (5 × 10^4^) were seeded in the upper chamber of cell culture inserts (8 µm pore size) in a 24-well plate in serum-free medium in triplicates. Complete medium was added as chemoattractant outside the inserts and the cells were incubated for 24 h at 37 °C and fixed in 4% (*w*/*v*) paraformaldehyde for 15 min and stained with 0.1% (*w*/*v*) crystal violet at RT for 15–20 min. The numbers of migrated cells on the lower surface of inserts were counted using a Nikon Eclipse TE2000-S microscope. 

### 2.13. RNA-Seq Analysis and Bioinformatics

Cells were plated in 10 cm dishes in duplicates. When they reached 80–90% confluence, the cells were washed twice with PBS and total RNA was extracted using Qiagen RNeasy kit (Cat #74104) according to manufacturer’s protocol. Samples were processed by Novogene Europe (Cambridge, UK) for library preparation and bioinformatics analyses. Quantified cDNA libraries were pooled and sequenced on high-throughput Illumina sequencing platform and paired-end reads were generated. Raw data (raw reads) of FASTQ format were firstly processed through *fastp* to clean the data by removing reads containing adapter, poly-N sequences and reads with low quality. Paired-end clean reads were aligned to the reference genome using *HISAT2* software. *Featurecounts* [61] was used to count the read numbers mapped to each gene, including known and novel genes. FPKM, (Fragments Per Kilobase of transcript sequence per Million base pairs sequenced) of each gene was calculated based on the length of the gene and reads count mapped to this gene. Differential expression analysis was performed using *DESeq2 R package*. The resulting *p* values were adjusted using the Benjamini and Hochberg’s approach for controlling the False Discovery Rate (FDR). Genes with an adjusted *p* value < 0.05 found by DESeq2 were assigned as differentially expressed. Gene Ontology (GO) enrichment and KEGG pathways analyses of differentially expressed genes was performed by the *clusterProfiler R package*. Corrected *p* value < 0.05 were considered significantly enriched by differentially expressed genes [62,63]. 

## 3. Statistical Analysis

All the experiments were performed in triplicates (technical repeats). To compare the two groups, two tailed Student’s *t*-tests were applied using Microsoft Excel and *p* values below 0.05 (*p* < 0.05) were considered significant. Linear regression analyses and Pearson correlation coefficients (Pearson’s r) were determined using data analysis tool ToolPak in Microsoft Excel. All results were represented as the mean of 3 individual experiments (n = 3) with standard error of the mean (±SEM). 

## 4. Results

### 4.1. Screening Human Cell Lines for Vimentin and Keratin K18 Expression

**Figure 1:**
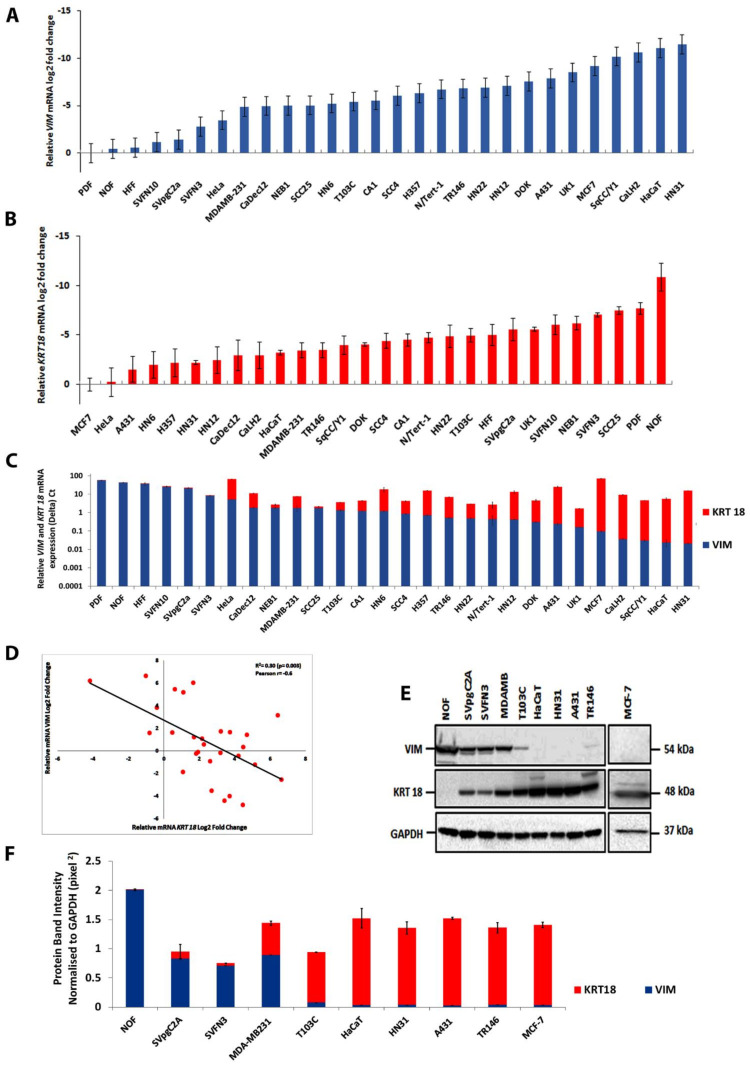
**Reciprocal relationship between vimentin and keratin K18 expression in tissue culture cell lines**. (**A**): The relative mRNA log2-fold change in 28 cell lines was determined by RT-qPCR. The relative *VIM* expression in primary dermal fibroblast (PDF) was taken as 100% and the percentage in other cell lines was calculated relative to it. The data were plotted in log2 format. The raw data for different cell lines are listed in Table S4. (**B**): The relative *KRT18* mRNA expression in MCF-7 was taken as 100% and the percentage in other cell lines was calculated relative to it. The data were plotted in log2 format. The raw data for different cell lines are listed in Table S5. (**C**): *VIM* and *KRT18* mRNA expression (ΔCt values) in different cell lines showing a reverse pattern of expression between two genes. (**D**) Regression analysis between *VIM* and *KRT18* expression showing modest inverse but significant correlation. (**E**): Vimentin and K18 expression in 10 different cell lines (NOF, SVpgC2A, SVFN3, MDA-MB-231, T103C, HaCaT, HN-31, A431, TR146 and MCF-7) by Western blotting. Fifty µg protein was loaded for each cell line using GAPDH as the loading control. Relevant bands were cropped from different gels and regrouped. Original blots are shown in Supplementary Figure S1 for vimentin and K18. (**F**): Quantification of vimentin and K18 bands using *ImageJ*. Statistical analyses: n = 3, Error bars = ±SEM.

To recapitulate the process of EMT in cancer cells where keratin expression is suppressed and vimentin expression is instigated, we first decided to identify cancer cell lines devoid of vimentin. We screened 28 different cancer cell lines plus normal epithelial and mesenchymal cells (Table S1) for the expression of *VIM* and *KRT18* (one of the major keratins in most cancer epithelial cells) by qPCR (Table S2 for qPCR primers). The results obtained for *VIM* were plotted with primary dermal fibroblasts (PDF) taken as 100% and calculating the expression in other cell lines relative to it (Table S4). Similarly, for *KRT18*, expression in MCF-7 was taken as 100% and the level in other cell lines was calculated accordingly (Table S5). The *VIM* expression relative to PDF, was highest in normal oral fibroblasts (NOF) followed by HFF, SVFN10, SVpgC2A, (Figure 1A). The *KRT18* expression in different cell lines relative to MCF7 was highest in HeLa, A431, HN31, HN12, H357, HaCaT, and CaLH2 (Figure 1B). We observed that VIM and K18 expression showed a reciprocal correlation (Figure 1C), which was statistically significant (*p* < 0.05) (Figure 1D). These results also showed that A431, UK1, MCF-7, SqCC/Y1, CaLH2, HaCaT and HN31 cell lines did not express any detectable *VIM* mRNA.

To corroborate the above data, we investigated vimentin and keratin K18 expression by Western blotting. As shown in Figure 1E the Western blotting data were consistent with the qPCR data in 10 different cell lines showing vimentin: K18 ratio was highest in NOF followed by SVpgC2A, SVFN3, MDA-MB-231, T103C, HaCaT, HN31, A431, TR146 and MCF-7 (Figure 1F). No specific vimentin band was detectable in HaCaT, HN31, A431 and MCF-7. A faint vimentin band was observed after a very long exposure (200s) in TR146 whereas no specific K18 band was detectable in NOF. 

### 4.2. Ectopic Expression of Vimentin in MCF-7 Cells Downregulated KRT8, KRT18 and KRT19

**Figure 2:**
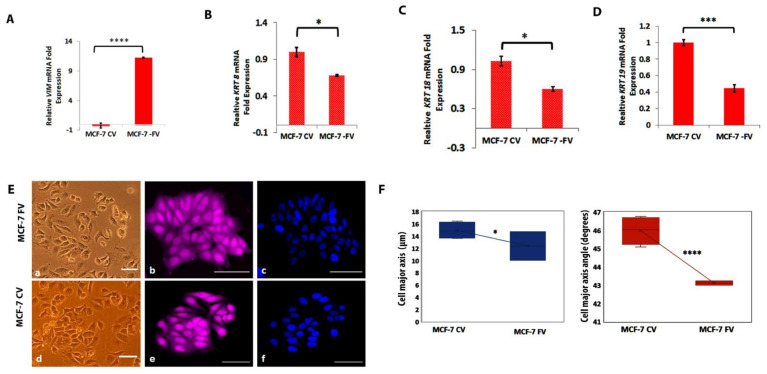

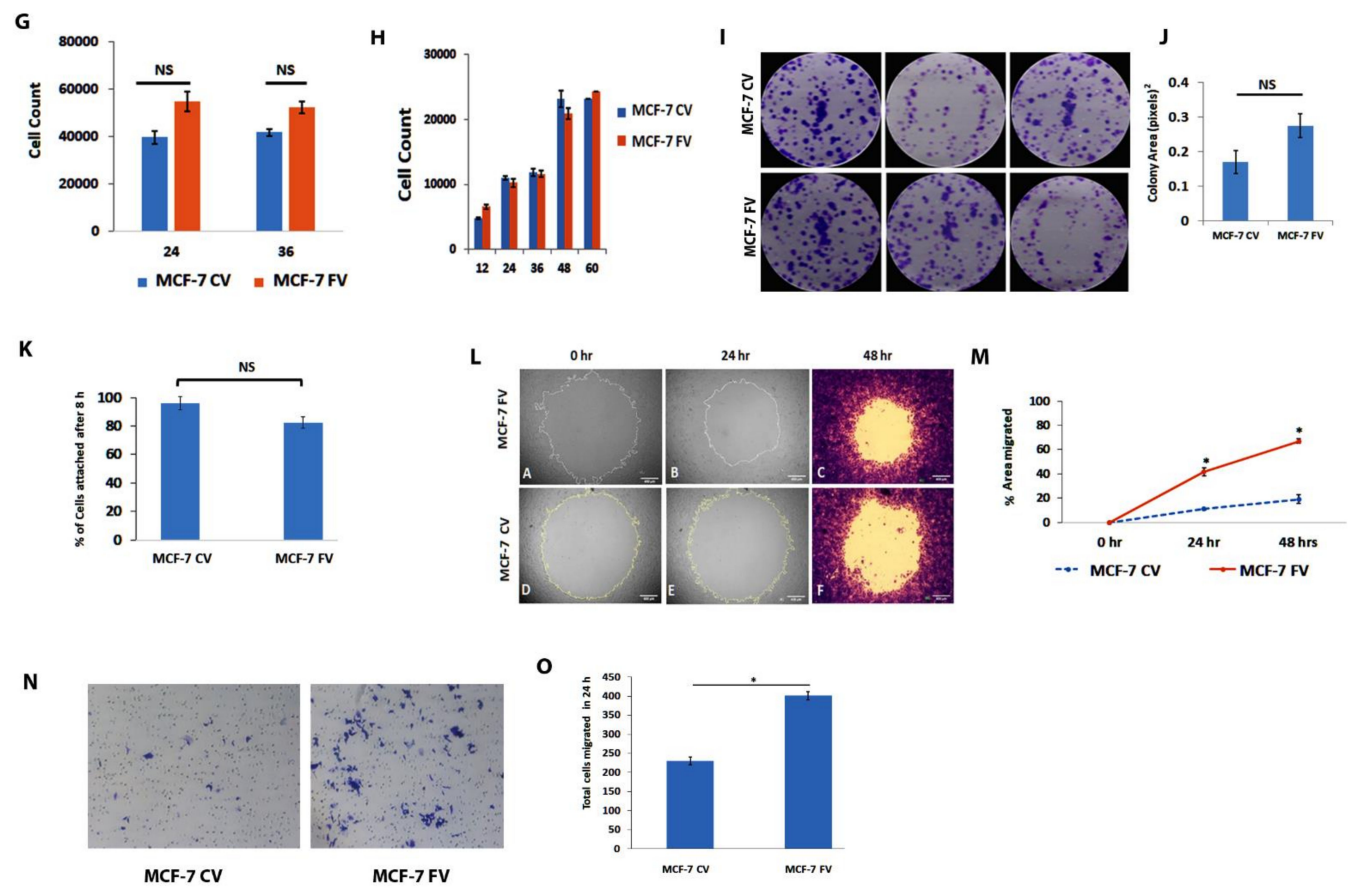
**Characterisation of vimentin-expressing MCF-7 cells.** Relative log2-fold expression of mRNA for *VIM* (**A**); *KRT 8* (**B**), *KRT 18* (**C**), *KRT19* (**D**) genes normalised to *POLR2A* and *YAP1* in MCF-7 expressing FV and CV. (**E**): Morphology of MCF-7FV (panel a, b) and MCF-7CV (panel d, e) in brightfield and Cellmask^TM^ deep red dye, respectively, captured by INCA 2200. Panel c and f shows DAPI staining. Scale bar = 20 μm (**F**): Significant differences in cell major axis (in µm) and major axis angle (in degrees) of MCF-7FV and MCF-7CV were observed using the INCA 2200 cell imaging system. Insignificant data are provided in Supplementary Figure S3. No significant changes (NS) were observed between proliferation of MCF-7FV and MCF-7CV using (**G**) CyQUANT and (**H**) MTT assays (**I**): Colony forming ability of MCF-7 cells (500 cells/well in 6 well plates in triplicate) expressing FV and CV were determined by growing them for 2 weeks in complete medium. Cells were fixed and stained with 0.1% (*w*/*v*) crystal violet. (**J**): Quantification of total area of MCF-7 FV and CV in pixel density calculated by *ImageJ*. (**K**): CyQUANT cell adhesion assay was performed to compare the adhesion between MCF-7FV and MCF-7CV cells. (**L**): Migration assays were performed using Oris^TM^ Cell Migration Assay kit for MCF-7FV and MCF-7CV cells at 24 h and 48 h according to manufacturer’s instructions. Scale bar = 200 μm (**M**): Percentage of the area migrated by MCF-7FV and CV was calculated by *ImageJ*. (**N**): Chemotactic migration in MCF-7FV and CV towards serum was determined by using 8.0 µm culture inserts in 24 well plates in triplicates. The cells were fixed and stained with 0.1% crystal violet. (**O**): The cells on the outer surface of the inserts were counted under the microscope. Statistical analyses: n = 3, Error bars = ±SEM, Student’s *t*-test was used to calculate *p* values using Microsoft Excel and are given by asterisks (* *p* < 0.05, *** *p* < 0.001 and **** *p* < 0.0001).

Based on the qPCR and Western blot analyses, we selected MCF-7, a simple epithelial breast carcinoma cell line devoid of endogenous vimentin to recapitulate the EMT process and ectopically expressed the full-length vimentin (pLPChygro-FV) or the control vector (pLPChygro-CV). The Western blot and immunofluorescence analyses of puromycin resistant clones confirmed that vimentin was expressed in MCF-7 at significantly higher (*p* < 0.0001) level (almost 100%) in MCF-7FV than in MCF-7CV when 10 or 20 µg protein was loaded confirming that the clones were stable (Figure S2A–D). We measured the mRNA expression for *VIM*, *KRT8*, *KRT18* and *KRT19* in these cells. The qPCR analysis showed that *VIM* expression in MCF-7FV cells was (11.23 ± 0.088 log2-fold) higher (*p* < 0.05) compared to MCF-7CV (Figure 2A). Interestingly, all the three keratin genes *KRT8* (0.68 ± 0.012 log2-fold), *KRT18* (0.59 ± 0.033 log2-fold) and *KRT19* (0.45 ± 0.044 log2-fold) were significantly downregulated (*p* < 0.05) in MCF-7FV cells suggesting that vimentin was able to reduce keratin gene expression (Figure 2B–D).

### 4.3. Ectopically Expressed Vimentin Alters MCF-7 Morphology

Next, to determine whether vimentin was also able to alter the cellular morphology, we fixed and stained the MCF-7FV and MCF-7CV cells with CellMask^TM^ Deep Red dye and DAPI as explained in the ‘Materials and Methods’ (Figure 2E). Different cell morphological features such as cell form factor, nuclear form factor, cell perimeter, cell diameter, nuclear area, nuclear/cell area, cell major axis, cell major axis angle, and cell minor axis were analysed. Nuclear to cell area was increased whereas cell form factor, nuclear form factor, nuclear area and cell perimeters were decreased in MCF-7FV but the changes were not significant (Figure S3). Significant reduction was observed in cell major axis angle (*p* < 0.0001) and cell major axis (*p* < 0.05) in MCF-7FV (Figure 2F).

### 4.4. Vimentin Does Not Change Cell Proliferation or Adhesion but Induces Cell Migration

To investigate whether vimentin affects cell proliferation, we performed CyQUANT (Figure 2G), MTT (Figure 2H) and colony formation assays (Figure 2I,J) in MCF-7FV and MCF-7CV. No significant difference in cell proliferation rate was observed in MCF-7FV compared with MCF-7CV. We also compared the cell adhesion of MCF-7FV with MCF-7CV by CyQUANT cell adhesion assay (Figure 2K). A decreasing trend in MCF-7FV was observed compared with MCF-7CV, however the difference was not significant (*p* > 0.05). We further investigated the effect of vimentin on cell migration using Oris^TM^ system. As shown in Figure 2L, migration was significantly increased (*p* < 0.05) in MCF-7FV as compared with MCF-7CV at 24 h and 48 h (Figure 2M). We also observed significantly increased migration (*p* < 0.05) of MCF-7FV cells through 8 µm pore size membrane towards FCS as chemoattractant compared with MCF-7CV (Figure 2N,O). 

### 4.5. RNA-Seq and Bioinformatics Analyses

**Figure 3:**
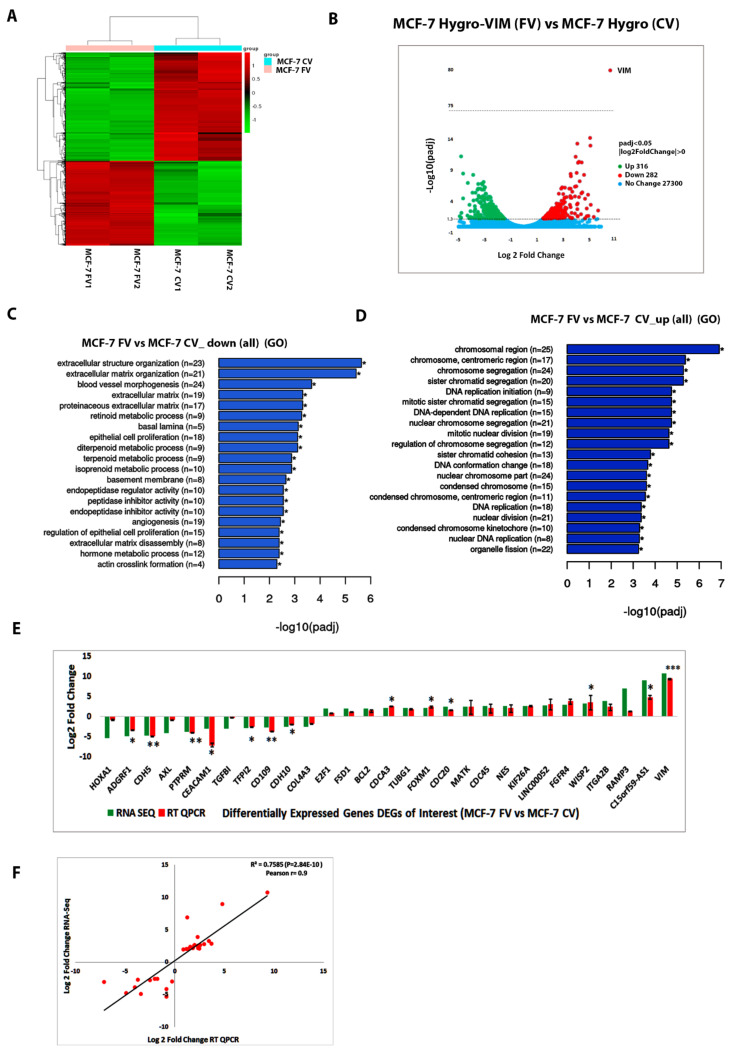
**Transcriptome analyses of MCF-7FV and CV cells**. (**A**): Heat map showing RNA-Seq transcriptome analysis in duplicate samples of MCF-7hygro-VIM (FV) and MCF-7hygro (CV) (**B**): Volcano plot showing the overlapping genes and DEGs between MCF-7FV and CV. The dotted line represents *p* = 0.05 [−log10 (1.30)] (**C**): Gene Ontology (GO) functional analysis showing combined overview of all cellular functions significantly downregulated by DEGs. (**D**): GO functional analysis showing combined overview of all cellular functions significantly upregulated by DEGs. (**E**): RNA-Seq analysis showing log2-fold expression of DEGs of interest and validation of RNA-Seq data by RT-qPCR for DEGs of interest. (**F**): Linear regression analysis of log2-fold changes calculated from qPCR and RNA-Seq analysis of DEGs. Red dots represent log2-fold change obtained from qPCR (X-axis) and RNA-Seq analysis (Y-axis) and the black line indicates a linear regression line. There was a highly significant (*p* < 0.00001) correlation (R^2^ = 0.75, Pearson r = 0.9) between the two data sets. Statistical analyses: n = 3, Error bars = ±SEM, Student’s *t*-test was used to calculate *p* values using Microsoft Excel. The *p* values are given by asterisks (* *p* < 0.05, ** *p* < 0.01, and *** *p* < 0.001).

As ectopic expression of vimentin in MCF-7 cells downregulated *KRT8*, *KRT18* and *KRT19* (Figure 2B–D) and also increased cell migration, we investigated the transcriptome profile of MCF-7FV using RNA-Seq in order to understand the underlying mechanism. Differential expression analysis showed that a total of 598 genes were differentially expressed (DEGs), out of which 282 (47.16%) were downregulated and 316 (52.8%) were upregulated (FDR < 0.05 and log2 FC > 0) (Figure 3A,B). RNA-Seq analysis showed a repressed trend in the *KRT8*, *KRT18* and *KRT19* expression in the presence of vimentin, however the data were not significant. In addition, transcriptome analysis revealed downregulation of several keratin genes but it was significant only for *KRT4* (Table S6). Detailed data sets for upregulated and downregulated genes are provided in Tables S7 and S8, respectively, and the list of upregulated and downregulated lncRNAs is given in Tables S9 and S10, respectively. 

### 4.6. Functional Enrichment Analysis of DEGs

To understand the overall functional implications of DEGs, Gene Ontology (GO) and the Kyoto Encyclopaedia of Genes and Genomes (KEGG) enrichment analyses were performed. Detailed functional implications of DEGs by GO analysis are explained in Figure S4. Each group was split into three major functional types: Molecular Function (MF), Biological Process (BP) and Cellular Component (CC) (Figure S4). Overall, the DEGs were classified into two major groups, the downregulated (Figure 3C) and upregulated (Figure 3D). The downregulated DEGs were mostly associated with cell–cell, cell-ECM interactions, angiogenesis and endopeptidase activities (Figure 3C and Figure S4), whereas the upregulated genes were mostly associated with cell proliferation such as DNA replication, chromosome segregation, chromosome condensation and organelle fission (Figure 3D and Figure S4). The data were further analysed by the KEGG pathway enrichment analysis which gave a very similar trend for downregulated (Figure S5A,B) as well as for upregulated genes (Figure S5C,D).

To validate the RNA-Seq data, we selected 28 DEGs (+*VIM*) of interest based upon log2-fold change in the genes associated with cell–cell adhesion, cell migration or proliferation. The expression profile of DEGs of interest by qPCR was consistent with the RNA-Seq data, confirming the reliability of the RNA-Seq analysis (Figure 3E). The extent of correlation of log2-fold change between RNA-Seq and RT-qPCR by regression and Pearson analyses gave a linear correlation (Figure 3F) with an R^2^ value of 0.7585 (*p* = 2.84 × 10^−10^) and Pearson coefficient of 0.9 suggesting that gene expression determined by RNA-Seq was highly consistent with the qPCR data.

### 4.7. shRNA Mediated Downregulation of Vimentin in MCF-7FV Suppressed Its Migration and Reversed the Profile of DEGs of Interest

**Figure 4:**
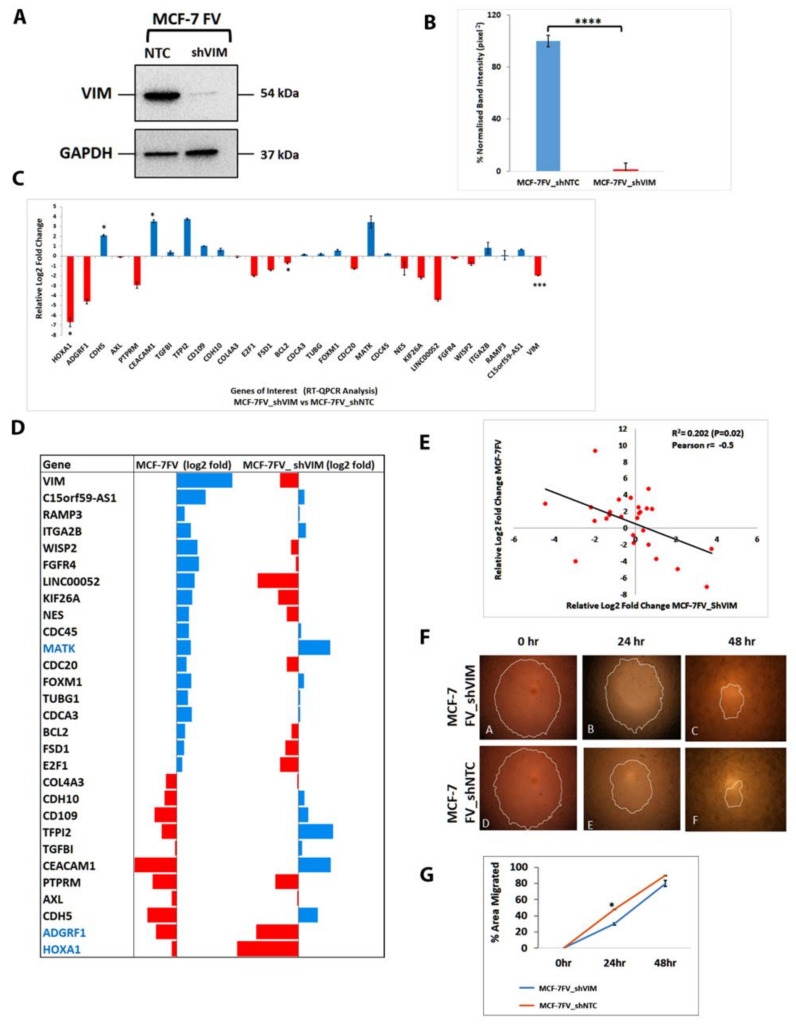
**Knockdown of vimentin in MCF-7FV by shRNA.** (**A**): Vimentin expression in MCF-7FV_shVIM and MCF-7FV_shNTC by Western blotting. Five µg protein was loaded for both the cell types. GAPDH was used as the loading control. Relevant bands were cropped from different gels and regrouped. Original blots are shown in Supplementary Figure S6 (**B**): Quantification of vimentin expression in MCF-7FV_shVIM and MCF-7FV_shNTC using *ImageJ*, Note the expression of vimentin was downregulated by 95% ± 3.6 in MCF-7FV_shVIM compared with MCF-7FV_shNTC. (**C**): Log2-fold change expression of DEGs of interest in MCF-7FV_shVIM relative to MCF-7FV_shNTC. (**D**): Comparison of patterns of log2-fold change of DEGs of interest in MCF-7FV and MCF-7FV_shVIM. Note the reverse expression pattern of all genes in MCF-7FV_shVIM as compared to MCF-7FV except for *MATK*, *ADGRF1* and *HOXA1*. (**E**): Inverse regression analysis of log2-fold changes between MCF-7FV_shVIM (X-axis) as compared with MCF-7FV (Y-axis). Red dots represent log2-fold change obtained from qPCR analysis and black line indicates an inverse regression. Note there was a significant (*p* < 0.05) but moderate inverse correlation (R^2^ = 0.202, Pearson r = −0.5) between the two data sets. (**F**): Representative images of cell migration assay in MCF-7FV_shVIM and MCF-7FV_shNTC at 0, 24, 48 h. (**G**): The line graph shows percentage area migrated by the cells at different time points. Note the decreased migration (significant at 24 h, *p* < 0.05) in MCF-7FV_shVIM as compared with MCF-7FV_shNTC. Statistical analyses: n = 3, Error bars = ±SEM, Student’s *t*-test was used to calculate *p* values using Microsoft Excel and are given by asterisks (* *p* < 0.05, *** *p* < 0.001 and **** *p* < 0.0001).

To determine whether vimentin in MCF-7 was responsible for the DEGs of interest, we downregulated vimentin in MCF-7FV cells by VIM-shRNA to more than 95% (*p* < 0.0001) as determined by Western blotting compared with the NTC (Figure 4A,B). The puromycin resistant cells were named MCF-7FV_shVIM. We measured the expression of 28 (+*VIM*) genes of interest by qPCR in MCF-7FV_shVIM and compared it with the NTC. Log2-fold change was significant (*p* < 0.05) in case of *VIM*, *BCL2*, *CEACAM1*, *CDH5* and *HOXA1* (Figure 4C). As shown in Figure 4D, Table S11 the expression profile of genes of interest reversed in MCF-7FV_shVIM cell line except for *HOXA1*, *ADGRF1* and *MATK* genes when compared with MCF-7FV. Regression analysis for the degree of correlation between genes of interest in MCF-7FV versus MCF-7FV_shVIM (Figure 4E) showed a reciprocal relationship with modest but significant (*p* < 0.05) correlation between the two sets. 

Next, we determined whether downregulating vimentin in MCF-7FV would reduce cell migration. Therefore, we compared migration of MCF-7FV_shVIM cells with MCF-FV_shNTC. As shown in Figure 4F, the migration was significantly reduced (*p* < 0.05) at 24 h in MCF-7FV_shVIM cells compared with NTC Figure 4G. However, this effect was not significant after 48 h which could be because the wound was almost closed after 48 h making this time point less reliable to compare the migration rates between the two cell types. 

### 4.8. Vimentin Downregulation in MDA-MB-231 Cells Induced Changes Similar to Those in MCF-7FV_shVIM

**Figure 5:**
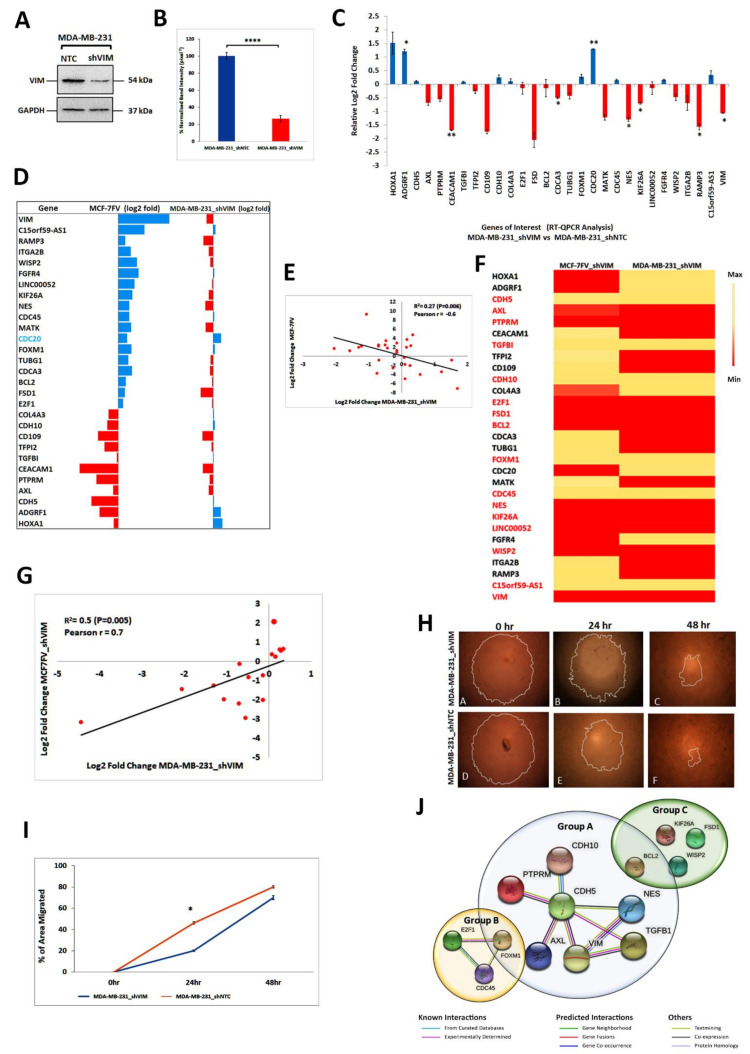
**Knockdown of vimentin in MDA-MB-231 cells by shRNA.** (**A**): Vimentin expression in MDA-MB-231_shVIM and MDA-MB-231_shNTC by Western blotting. Five µg protein was loaded for both cell lines and GAPDH was used as loading control. Relevant bands were cropped from different gels and regrouped. Original blots are shown in Supplementary Figure S6. (**B**): Quantification of vimentin expression in MDA-MB-231_shVIM and MDA-MB-231_shNTC was performed using *ImageJ*. (**C**): DEGs of interest log2-fold change expression in MDA-MB-231_shVIM relative to MDA-MB-231_shNTC. (**D**) Comparison of patterns of log2-fold change of DEGs of interest in MCF-7FV and MDA-MB-231_shVIM. The pattern of all genes in MDA-MB-231_shVIM as compared to MCF-7FV was reversed except for *CDC20*. (**E**): Regression analysis of log2-fold change between MDA-MB-231_shVIM (X-axis) as compared to MCF-7FV (Y-axis). Red dots represent log2-fold change values obtained from qPCR analysis and black line indicates a linear regression line. There was significant (*p* = 0.006) but moderate inverse correlation (R^2^ = 0.27, Pearson r = −0.6) between the two data sets. (**F**): Heatmap showing DEGs (log2-fold change) of interest comparison between MCF-7FV_shVIM and MDA-MB-231_shVIM. Red colour indicates the values in lowest log2-fold and yellow colour indicates the values in highest log2 fold. Sixteen out of 29 DEGs of interests were expressing the same pattern of log2-fold changes and 2/16 were lncRNA (**G**): Regression analysis of log2-fold changes between MDA-MB-23_shVIM (X-axis) and MCF-7FV_shVIM (Y-axis) of 16 genes selected on the basis of the heatmap in panel F. Red dots represent log2-fold change obtained from qPCR analysis and black line indicates a linear regression line. There was a significant (*p* = 0.005) positive correlation (R^2^ = 0.5, Pearson r = 0.7) between the two data sets. (**H**): Representative images of migration of MDA-MB-231_shVIM and MDA-MB-231_shNTC at 0, 24, 48 h. (**I**): The percentage area migrated by the cells at different time points decreased in MDA-MB-231_shVIM compared to NTC control. (**J**): Protein–Protein interactions using STRING analysis groups the 14 DEGs of interest into two main functions, maintaining cell–cell junctions (Group A) and cell proliferation (Group B). Four proteins remained with undefined function (Group C). Inclusion of FOXM1 and BCL2 in group A can be justified based on published data (see text for details). Statistical analyses: n = 3, Error bars = ±SEM, Student’s *t*-test was used to calculate *p* values using Microsoft Excel and are given by asterisks (* *p* < 0.05, ** *p* < 0.01, and **** *p* < 0.0001).

To corroborate the above findings, we downregulated endogenous vimentin in MDA-MB-231 cells, a vimentin-expressing breast cancer cell line [64], by VIM-shRNA. The puromycin resistant cell line was named MDA-MB-231_shVIM and the corresponding control MDA-MB-231_shNTC. Western blotting confirmed that vimentin expression was reduced by 75% (*p* < 0.0001) in MDA-MB-231_shVIM compared with NTC (Figure 5A,B and Figure S6). The qPCR analysis confirmed significant (*p* < 0.05) downregulation of the *VIM*, *RAMP3*, *KIF26A*, *NES*, *CDCA3*, *CEACAM1*, compared with NTC, whereas significant (*p* < 0.05) upregulation was seen in the case of *CDC20* and *ADGRF1* (Figure 5C). Comparison of DEGs trend between MDA-MB-231_shVIM with MCF-7FV indicated that except for *CDC20*, the expression of all the genes of interest was reversed (Figure 5D, Table S12). The regression analysis of log2-fold change between MCF-7FV and MDA-MB-231_shVIM showed an inverse significant correlation with R^2^ = 0.27 (*p* = 0.006) and modest Pearson coefficient (r = −0.6) (Figure 5E). 

### 4.9. Vimentin Responsive Genes Common in MCF-7FV_shVIM and MDA-MB-231_shVIM

Our data suggested that downregulation of vimentin in breast cancer cell lines either expressing vimentin endogenously (MDA-MB-231) or ectopically (MCF-7FV), had a common set of DEGs (Figure 5F, Table S13). The DEGs that showed a similar pattern in both systems that was also reversed in MCF-7FV include *CDH5*, *AXL*, *PTPRM*, *TGFBI*, *CDH10*, *E2F1*, *FSD1*, *BCL2*, *FOXM1*, *CDC45*, *NES*, *KIF26A*, *WISP2*, *LINC00052* and *C15ORF9-AS1* suggesting they represented a common vimentin-responsive gene signature. Regression analysis (Figure 5G) showed a modest (R^2^ = 0.5) but significant (*p* = 0.005; Pearson r = 0.7) linear correlation between log2-Fold change in MCF-7FV_shVIM and in MDA-MB-231_shVIM cells. We further investigated the effect of vimentin downregulation in MDA-MB-231 on cell migration and as shown in Figure 5H,I, migration of MDA-MB-231_shVIM was significantly reduced as compared to NTC (*p* < 0.05 at 24 h) suggesting that the endogenously expressed vimentin was also able to increase cell migration. Similar to the trend in MCF-7FV_shVIM (Figure 4G), this effect was not significant after 48 h. 

To understand the biological processes regulated by vimentin responsive 15 genes identified above, we used STRING Protein–Protein Interaction Networks Functional Enrichment Analysis to predict interactions between their gene products. We used only 13 genes as two of them, *LINC00052* and *C15ORF9-AS1*, were lncRNAs. As shown in Figure 5J STRING was able to segregate the 13 proteins into 3 groups, group A contained 6 proteins, CDH5, AXL, PTPRM, TGFBI, CDH10, NES and most of them were linked with either cell–cell or cell-ECM association. Group B had only three proteins, CDC45, E2F1 and FOXM1 and they were associated with cell proliferation. Group C contained the remaining 4 proteins, WISP2, BCL2, KIF26A and FSD1 and they did not show any association amongst themselves or with vimentin. 

### 4.10. Downregulation of CDH5 in MCF-7 Increases Cell Migration

**Figure 6:**
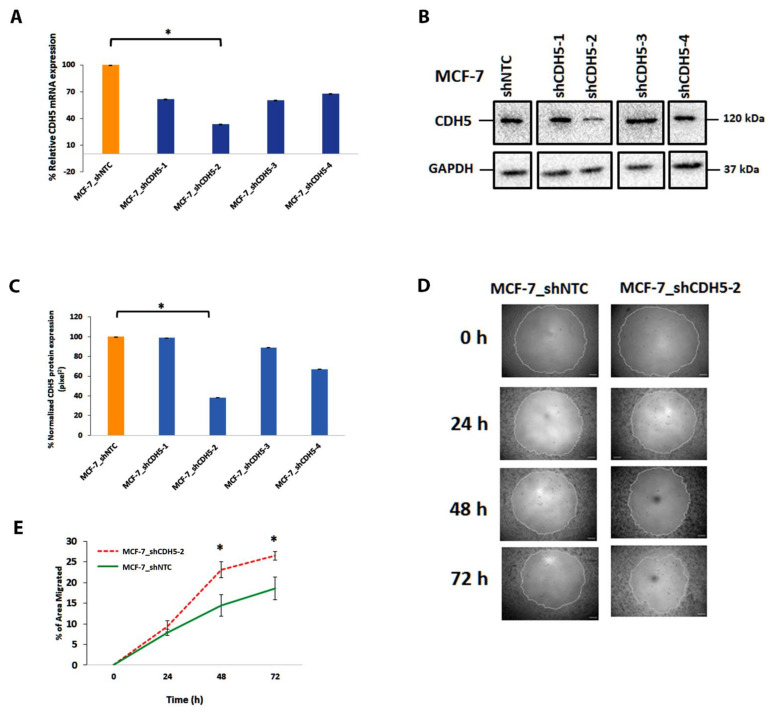
**CDH5 knockdown increases MCF-7 migration:** (**A**) MCF-7 cells were transduced with recombinant retroviruses expressing 4 different shRNAs (sh1-sh4) for CDH5 or NTC and relative CDH5 mRNA expression was determined by qPCR. Maximum and significant downregulation (*p* < 0.01) was observed in MCF-7 with shCDH5-2 as compared with shNTC. (**B**): CDH5 expression in the MCF-7 transduced with shCDH5-2 or shNTC retrovirus by Western blotting. A total 5 μg protein was loaded for each cell type and probed with anti-CDH5 antibody (1:1000) dilution. GAPDH was used as the loading control. Original blots are shown in Supplementary Figure S7. (**C**): Quantification of the CDH5 bands in panel (**B**) was carried out by *ImageJ*, significant downregulation (*p* < 0.05) of CDH5 in MCF-7 transduced with the second shRNA construct producing MCF-7_shCDH5-2 cells as compared to shNTC that corroborated qPCR data (panel (**A**)). (**D**): Representative images of cell migration assay in MCF-7_shCDH5-2 and shNTC at 0, 24, 48 and 72 h. (**E**): Graph showing percentage area migrated by the cells at different time points and a significant (*p* < 0.05) increase in migration of MCF-7_shCDH5-2 at 48 and 72 h was observed. Statistical analyses: n = 3, Error bars = ±SEM, Student’s *t*-test was performed to calculate *p* values using Microsoft Excel and are given by asterisks (* *p* < 0.05).

As ectopic vimentin expression in MCF-7 particularly downregulated the genes associated with cell–cell junctions, and the STRING analysis showed that CDH5, which assembles adherens junctions in endothelial cells, showed direct interactions with 6 proteins, 4 of which had been experimentally determined (Figure 5J), we argued that knocking down CDH5 should mimic the effect of vimentin expression. Using shRNA, we knocked down CDH5 in MCF-7 cells and confirmed significant knockdown (*p* < 0.05) in MCF-7_shCDH5-2 cells by qPCR (Figure 6A) and also by Western blotting (Figure 6B,C). Cell migration was also significantly increased (*p* < 0.05) at 24 and 48 h (Figure 6D,E) in MCF-7_shCDH5-2 compared with NTC. These results suggested that downregulation of CDH5 weakens the cell–cell adhesion and consequently increases cell migration. As expression of vimentin and CDH5 are inversely related, vimentin might be increasing the cell migration by downregulating transcription of genes associated with cell–cell junctions. 

### 4.11. Vimentin Expression in MCF-7 Alters the Lamin Expression

**Figure 7:**
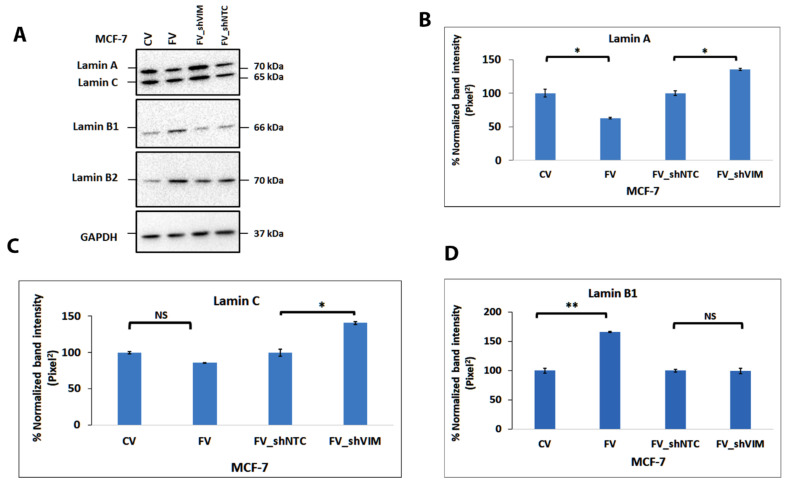

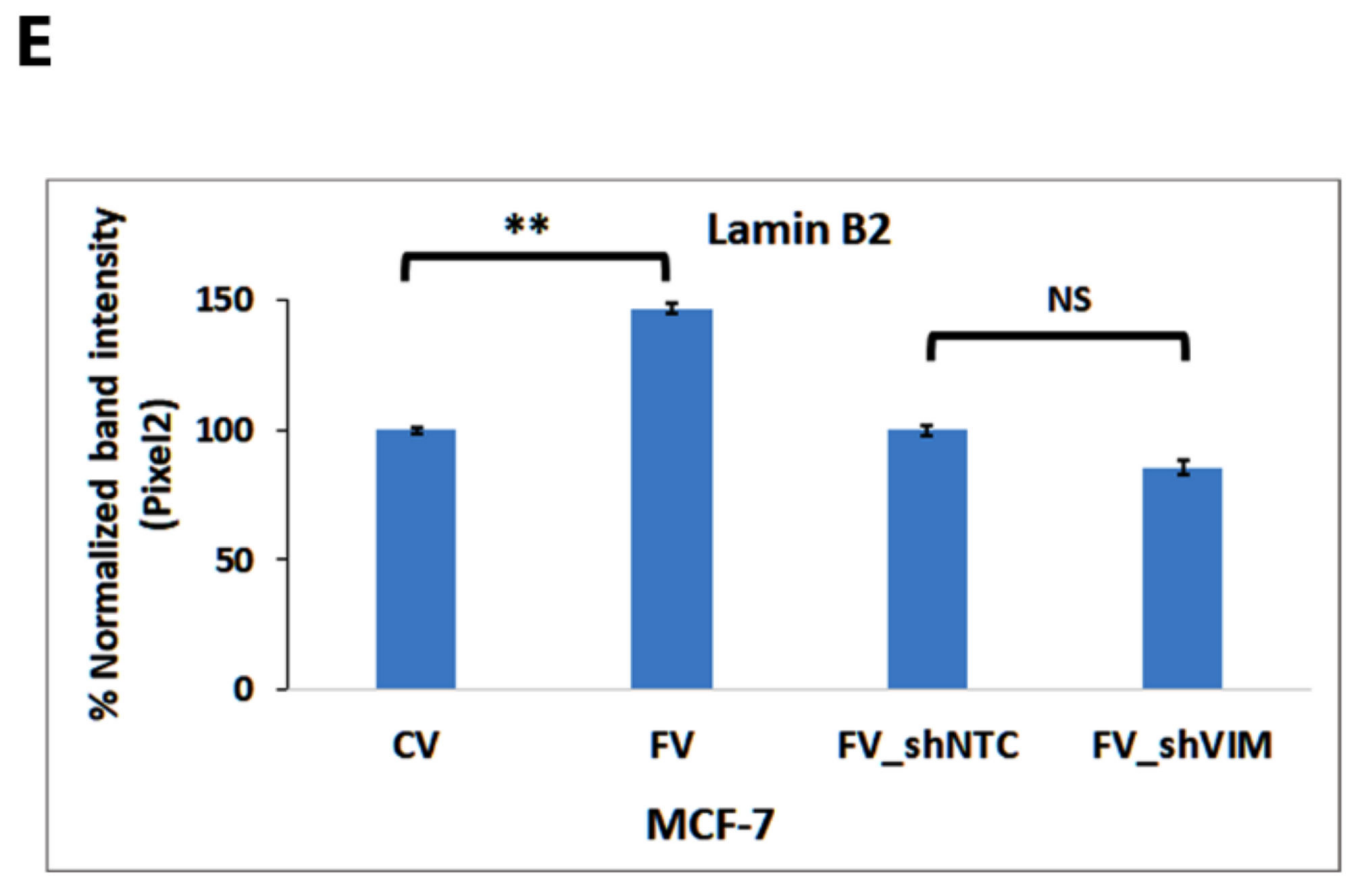
**Effect of vimentin on the expression of nuclear lamins in MCF-7 cells.** (**A**): Lamin expression in MCF-7CV, MCF-7FV cells and after transduction of MCF-7FV with shVIM and NTC to produce MCF-7FV_shVIM and MCF-7_shNTC cells was determined by Western blotting. A total of 10 μg protein was loaded for each cell line and probed with anti-lamin A/C, B1 and B2 antibodies. GAPDH was used as the loading control. Relevant bands were cropped from different gels and regrouped. Original blots are shown in Supplementary Figure S8. (**B**): Quantification of the band intensity of lamin A indicated significant downregulation (*p* < 0.05) in MCF-7FV as compared to CV whereas significant upregulation (*p* < 0.05) was seen in MCF-7FV_shVIM as compared to MCF-7_shNTC. (**C**): Quantification of the band intensity in case of lamin C indicated a repressed trend however this change was not significant (NS) between CV and FV. The pattern was significantly (*p* < 0.05) reversed in MCF-7_shVIM as compared to shNTC. (**D**): Quantification of the band intensity in case of lamin B1 showed significant upregulation (*p* < 0.01) in MCF-7FV as compared to CV whereas this trend was not reversed by specific shRNA. (**E**): Quantification of the protein bands in case of lamin B2 indicated significant upregulation (*p* < 0.01) in MCF-7FV as compared to CV whereas no significant change was seen in MCF-7FV_shVIM as compared to MCF-7_shNTC. Statistical analyses: n = 3, Error bars = ±SEM, Student’s *t*-test was performed to calculate *p* values using Microsoft Excel and are given by asterisks (* *p* < 0.05, ** *p* < 0.01, NS = Not significant).

To provide insight into the possible mechanism of transcriptional regulation of DEGs by vimentin and its effect on cell migration, we hypothesized that vimentin was affecting the transcriptional regulation through lamin expression. To test this hypothesis, we compared lamin expression in MCF-7FV with that in MCF-7CV by Western blotting. This was logical as vimentin and lamins are both part of the LINC complex, which is involved in gene transcription [24,25]. Interestingly, the expression of lamin A was significantly (*p* < 0.01) downregulated but lamin B1 and 2 were significantly upregulated (*p* < 0.001) in MCF-7FV compared with MCF-7CV (Figure 7A,D,E). A decreasing non-significant trend of lamin C was observed in MCF-7FV compared to MCF-7CV (Figure 7C). Similar trends of lamin expression were observed in RNA-Seq analysis but the data were not significant (Table S6). The reduced expression of lamin A and C was reversed (Figure 7B,C) after vimentin downregulation in MCF-7FV_shVIM indicating a direct effect of vimentin. However, no significant reversibility in expression was observed in lamin B1 and B2, perhaps suggesting an indirect effect (Figure 7D,E). 

## 5. Discussion

During malignant progression, epithelial cells undergo EMT in which keratin expression is reduced and vimentin expression is increased [65]. Previously, we have shown that downregulation of vimentin in HN12 cells, a head and neck cancer cell line derived from a metastatic tumor deposit, induces keratin promoter activity and enhances keratin expression [30]. Furthermore, in the current study, we have demonstrated a reciprocal relationship between keratin K18 and vimentin expression in 28 cell lines, most of them expressing both vimentin and keratin K18, and several of them (A431, UK1, MCF-7, SqCC/Y1, CaLH2, HaCaT and HN31) being deficient of vimentin. The reciprocal relationship between vimentin and keratin was further demonstrated by ectopic expression of full-length vimentin into MCF-7 cells which led to suppression of *KRT4*, *KRT8, K18* and *KRT19* expression. This observation is consistent with other in vitro studies [27] and also in breast cancer patients where a high vimentin/keratin ratio was associated with poor prognosis [66]. 

The cell shape measurements of MCF-7FV cells compared to empty vector-transfected cells showed that changes in cell or nuclear form factor (i.e., how round the cells were), cell perimeter, cell to nuclear area and other cell morphological characteristics were insignificantly different between the two cell types. However, major axis length and major axis angle were significantly reduced, suggesting that vimentin had made MCF-7 cells less elongated. These results are in contrast to previous reports showing that vimentin in cancer cells induces them to adopt mesenchymal features [28] although it does not affect embryonic stem cell morphology [67]. 

There are conflicting reports on the effect of vimentin on cell proliferation. While we found that vimentin does not influence proliferation of MCF-7 (Figure 2), which is consistent with previous reports [27], others have reported decrease in proliferation and invasive potential by vimentin in HepG2 [68]. Several studies have shown that vimentin increases cell proliferation [4,30]. Although the basis for these contradictory observations is not known, they may reflect the cell types used in different studies. 

Previous studies have shown that vimentin regulates cell-ECM association by controlling the size of focal adhesions through interacting with integrin β1 and β3 [69], vimentin also increases lung cancer cell adhesion by stabilising FAK-positive focal adhesions through activating Rac1 [70]. These studies suggest formation of larger FAs in presence of vimentin would not increase the number of cells adhering the substratum. This is consistent with our observation that the number of MCF-7 cells attached to the substratum does not change in the presence of vimentin. Similar observations have been reported previously for SW480 cells [71].

In this study, ectopic expression of vimentin induced migration of MCF-7 cells in both wound healing and chemotactic assays (Figure 2). Downregulation of vimentin in both MCF-7FV and in MDA-MB-231 suppressed cell migration, suggesting that vimentin plays a fundamental role in enhancing cell migration. These data are consistent with previous reports [28,31]. For example, we have reported previously that migration of HN12, a vimentin-expressing cell line, was increased in the presence of vimentin [30]. In our data, downregulation of vimentin in MCF-7FV and in MDA-MB-231 by shRNA reduced the cell migration significantly up to 24 h but this suppressed migration was not upheld after 48 h. A possible explanation can be that after 48 h wound was almost closed, so the measurement may not be reliable enough to distinguish changes in the migration rates. Taken together, these results indicate that vimentin-mediated cell migration is independent of cell type.

Several mechanisms have been proposed to explain vimentin induced cell migration. These include interaction of keratin and vimentin IFs at FA [72], upregulation of the receptor tyrosine kinase Axl [29], inducing collagen 1 synthesis by stabilizing its mRNA [73], assembly and regulation of FA size [74,75], and through a number of signalling pathways including Rac1/RhoA [76], NOTCH/ Jagged1 [77] and ERK/AKT/Rho1 [78]. If vimentin induces cell migration through regulating FA or modifying signaling cascades, one would expect migration of individual cells to be increased. Indeed, N-terminally tagged vimentin has been shown to increase migration of MCF-7 cells [4]. These data contradicted the report that vimentin was able to increase migration of MDA-MB-231 cells only in dense cultures [27]. This may indicate that vimentin does not increase cell migration through a direct mechanism. Although it is not clear how Liu and co-workers observed the effect of vimentin at individual cell level [4], it could be due to the tag used in their study. In this regard, we have recently shown that the N-terminally tagged vimentin only formed aggregates that aligned along intercellular junctions in an epithelial colony, and although the tagged vimentin integrated into the pre-existing network, the filaments were found to be weaker [55]. 

To understand the mechanism of vimentin-induced cell migration, we performed transcriptome profiling of MCF-7FV using RNA-Seq (Figure 3B). These data were validated with qPCR analyses and the regression analysis showed significant correlation between the two data sets with *p* value = 0.9. The overall functional analysis using both GO and KEGG pathways indicated that genes associated with cell–cell interactions and cell-ECM interactions were downregulated while genes associated with cell proliferation, cell cycle regulation and DNA replication were upregulated (Figure 3). From the GO and KEGG pathway functional analysis we selected 28 DEGs of interest (excluding VIM) and, to identify a vimentin-specific gene signature, we downregulated vimentin in MCF-7FV (ectopic VIM). Expression of all genes except for *MATK*, *ADGRF1* and *HOXA1* was reversed (Figure 4D; Table S11). The correlation analysis between MCF-7FV and MCF-7FV_shVIM showed an inverse correlation between the two data sets with *p* value = −0.5. Similarly, downregulation of vimentin in MDA-MB-231 (endogenous VIM) reversed expression of all the DEGs except for *CDC20* (Figure 5D; Table S12). The correlation analysis between MCF-7FV and MDA-MB-231_shVIM showed moderate inverse correlation between the two data sets with *p* value = −0.6. It is not clear why expression of some genes was not reversed. Comparison of the DEGs expression pattern in both MCF-7FV_shVIM and MDA-MB-231_shVIM produced a list of 15 genes—*CDH5*, *AXL*, *PTPRM*, *TGFBI*, *CDH10*, *E2F1*, *FSD1*, *BCL2*, *FOXM1*, *CDC45*, *NES*, *KIF26A*, *WISP2*, *LINC00052* and *C15ORF9-AS1*—showing similar patterns of expression that is reverse/opposite to that in MCF-7FV. The correlation analysis between MCF-7FV_shVIM and MDA-MB-231_shVIM showed positive correlation between the two data sets with *p* value = 0.7. The above-described gene list represented the common vimentin-responsive gene signature in ectopic and endogenous vimentin expressing cell systems. 

Out of the 15 vimentin-responsive genes, two were lncRNAs, *LINC00052* and *C15orf59-AS1*. Previous reports have suggested that *LINC00052* is linked to EMT, cancer cell migration, progression and invasion in hepatocellular and head and neck carcinoma [79]. This is the first study where vimentin is linked to upregulation of *LINC00052* expression in a cell culture model. *C15orf59-AS1* is the anti-sense RNA of *C15orf59* (also known as *INSYN1*) gene and there are no reports of its expression in MCF-7 or any association with cancer or vimentin expression. In this study we show that the expression of *C15orf59-AS1* in MCF-7 is vimentin-dependent. Overall, the vimentin-dependent gene signature, common between MCF-7FV_shVIM and MDA-MB-231_shVIM, suggests that vimentin contributes to aberrant expression of genes associated with intercellular junctions, migration, EMT, cell cycle and apoptosis. 

Analysis of Protein–Protein Interaction Networks Functional Enrichment Analysis using STRING showed that the products of 6 out 13 genes (*CDH5*, *AXL*, *PTPRM*, *TGFBI*, *CDH10* and *NES*) interacted directly or indirectly with vimentin and were associated with cell–cell and cell-ECM interactions (Group A). The proteins in the other group (FOXM1, E2F1 and CDC45) were associated with cell proliferation, DNA replication and cell cycle progression (Group B). Interestingly, FOXM1 is shown to regulate reannealing of endothelial adherens junction [80] and therefore can be part of both A and B groups. Furthermore, upregulation of BCL2 decreases cadherin mediated cell–cell adhesion [81], which would justify its inclusion in Group A. Therefore, 8 out of 13 proteins (CDH5, AXL, PTPRM, TGFBI, CDH10, NES, FOXM1 and BCL2) are associated with regulating cell–cell junctions with most interactions involving CDH5. As vimentin and *CDH5* gene expression was inversely related in our transcriptome analysis (Figure 3E and Figure 4C), we hypothesised that vimentin upregulates cell migration by downregulating transcription of the genes associated with cell–cell adhesion. Consistent with this hypothesis was our observation that downregulation of *CDH5* in MCF-7 increased cell migration (Figure 6). In a recent study Sivagurunathan and co-workers compared transcriptome profile of MCF-7 expressing vimentin filaments with those expressing only ULFs using a 4-isopropyl benzoate (cumate)-inducible system [82]. Their conclusion that filamentous vimentin suppresses formation of intercellular junctions thereby augments cell migration was based on the internalisation of desmoplakin, a component of desmosome which does not directly links adjacent cells. In contrast, we have shown that vimentin suppresses expression of genes associated with cell–cell junctions, the most prominent one is *CDH5*, which is an endothelial cadherin directly linking adjacent cells. Furthermore, Sivagurunathan et al. only analysed the genomic effects of transduced vimentin in MCF-7 cells, however, their DEGs set contained genes related to cell migration, upregulation of EMT transcription factors, cell adhesion/invasion, mesenchymal features and no effect on keratin gene expression. In our RNA-Seq data, we have observed downregulation of keratin genes and no upregulation of transcription factors TCF7, TWIST1 or mesenchymal markers. While the exact reasons for these differences are not clear, it may reflect the use of inducible system and ULF forming mutant (VIM^Y117L^) in the previous study [82]. 

**Figure 8:**
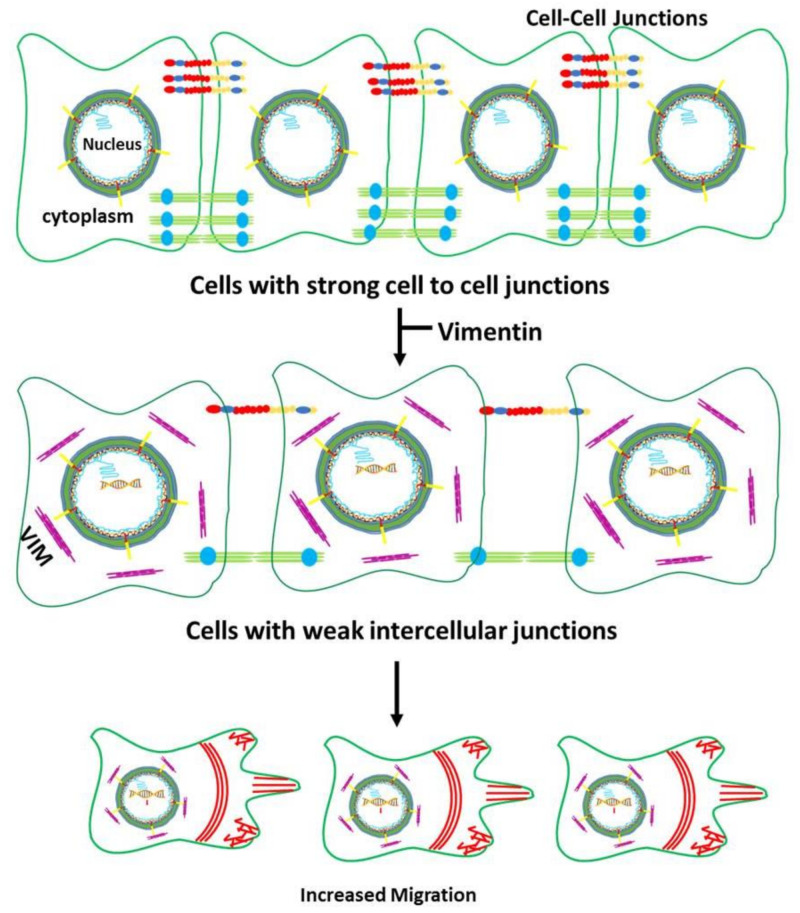
**A model for vimentin-induced augmentation of cell migration:** Expression of vimentin in breast cancer cells perturbs expression of lamins A, B1 and B2 which is predicted to have a subtle influence on the nuclear architecture as lamins are known to stabilize the nuclear lamina. This leads to changes in gene transcription, especially downregulation of those encoding cell–cell junction proteins (depicted in red/yellow/blue and green/blue lines). Consequently, this reduces intercellular associations and makes the cancer cells less adherent to each other, thus enhancing migration.

To explain the downregulation of cell–cell junction associated genes, we hypothesised that vimentin could suppress transcription of genes stabilising cell–cell junctions through its interaction with lamins (Figure 8). This premise was based on the fact that vimentin is part of the LINC complex, thereby linking the cytoskeleton with the nucleus [83]. Consistent with this hypothesis was the observation that lamin A was significantly downregulated (*p* < 0.01) in MCF-7FV which was reversed in MCF-7FV_shVIM, indicating a direct influence of vimentin on lamin A. However, lamin B1 and B2 were significantly (*p* < 0.001) upregulated in MCF-7FV, although the pattern was not reversed by shRNA (Figure 7D,E). The differential response between A and B lamins can be explained by the two types of lamins interacting differently with the LINC complex, as has been reported recently [26]. Consistent with the vimentin-induced altered levels of lamins, nuclear area was reduced, although not significantly, in MCF-7FV cells (Figure S3). Dysregulated transcription and chromatin reorganisation by mutant lamins is associated with a large number of laminopathies such as progeria, cardiomyopathy and muscular dystrophies; also, changes in lamin expression are associated with many cancers [84,85,86], and therefore it is plausible that vimentin-induced changes in lamin expression could suppress genes associated with cell–cell junctions. In contrast to our data, there are reports demonstrating that vimentin expression does not alter lamin expression [87,88]. However, these studies were conducted on murine embryonic fibroblasts (mEF) which are different from the vimentin-deficient epithelial cancer cells we have used in our study. Thus, it is conceivable that vimentin-mediated effects on lamin expression are associated with EMT and therefore absent in fibroblasts as they are already of mesenchymal origin. A limitation of this study is that the altered lamin expression induced by vimentin was not corroborated by the use of shRNA or similar approaches to inhibit lamin expression and study its effect on the nuclear architecture or on the expression of cell–cell junctional proteins and cell migration.

In conclusion, we have shown that vimentin downregulated keratin expression and, by implication, suppressed differentiation, thereby enhancing cancer progression and metastasis. The vimentin responsive genes could be segregated into two groups: those influencing cell–cell interactions and those affecting cell proliferation. The cell proliferation signal remained silent as vimentin did not influence cell proliferation. However, vimentin suppressed expression of genes associated with intercellular junctions and cell-ECM interactions, thereby weakening the cell–cell associations. Interestingly, vimentin might possibly modulate gene transcription by altering lamin expression. How changes in lamin expression downregulate genes associated with cell–cell junctions need further evaluation. Our study provides further insights into the important role of vimentin in EMT and metastasis by identifying downstream targets that could lead to development of novel strategies for therapeutic purposes. 

## Figures and Tables

**Table 1:** Transduced cell lines used in the study.

Cell Line	Insert	Drug Selection	Retrovirus Used
MCF-7CV	Control vector	Hygromycin	pLPChygro
MCF-7FV	Full-length vimentin cDNA	Hygromycin	pLPChygro-VIM
MCF-7FV_shNTC	Non-target control	Puromycin	shRNA-NTC
MCF-7FV_shVIM	Vimentin specific short hairpin	Puromycin	shRNA-VIM
MDA-MB-231_shNTC	Non-target control	Puromycin	shRNA-NTC
MDA-MB-231_shVIM	Vimentin specific short hairpin	Puromycin	shRNA-VIM
MCF-7_shNTC	Non-target control	Puromycin	shRNA-NTC for CDH5 knockdown
MCF-7_shCDH5-1	CDH5 specific short hairpin 1	Puromycin	shRNA-CDH5-1
MCF-7_shCDH5-2	CDH5 specific short hairpin 2	Puromycin	shRNA-CDH5-2
MCF-7_shCDH5-3	CDH5 specific short hairpin 3	Puromycin	shRNA-CDH5-3
MCF-7_shCDH5-4	CDH5 specific short hairpin 4	Puromycin	shRNA-CDH5-4

## Data Availability

All DEGs files and data are provided with this manuscript Supplementary File.

## Supplementary Materials

The following supporting information can be downloaded at: https://www.mdpi.com/article/10.3390/cells11244035/s1, Figure S1: VIM and KRT 18 expression in different cell lines; Figure S2: Ectopic expression of vimentin in MCF-7 cells; Figure S3: Morphology of MCF-7 expressing full-length vimentin; Figure S4: GO pathway enrichment analyses of DEGs by RNA-Seq; Figure S5: KEGG pathway enrichment analyses of DEGs by RNA-Seq; Figure S6: Protein expression in MDA-MB-231 and MCF-7FV cell lines transduced with shVIM and shNTC retroviruses; Figure S7: CDH5 expression in MCF-7 cell lines transduced with shRNA-CDH5 and shNTC retroviruses; Figure S8: Lamin A/C, B1 and B2 expression in MCF-7 cell lines transduced with FV, CV, shVIM and shNTC retroviruses; Table S1: Cell lines used in the study; Table S2: qPCR primers used in this study; Table S3: Primers used for cloning shRNAs; Table S4: Relative VIM mRNA fold change expression using qRT-PCR in 28 different cell lines normalized to PDF; Table S5: Relative KRT18 mRNA fold change expression using qRT-PCR in 28 different cell lines normalized to MCF-7; Table S6: Log2fold-change in expression of different keratins and lamins in MCF-7FV vs MCF-7CV; Table S7: List of upregulated DEGs by RNA-Seq analysis (Log2 Fold Chang >3.01); Table S8: List of downregulated DEGs by RNA-Seq analysis (Log2 Fold Change range from −6 to −3); Table S9: List of upregulated long non-coding RNAs using RNA-Seq analysis (Log2 Fold Change >1.7); Table S10: List of downregulated long non-coding RNAs using RNA-Seq analysis (Log2 Fold Change range from −6 to 0.0008); Table S11: Comparison of relative log2fold changes in DEGs of interest between MCF-7FV and MCF-7FV_shVIM; Table S12: Comparison of relative log2fold changes in DEGs of interest between MCF-7FV and MDA-MB-231_shVIM; Table S13: Heatmap generated by comparison of relative log2fold- change of genes of interest in MCF-7FV_shVIM and MDA-MB-231_shVIM; DEGs and Functional analysis files. References [32,33,34,35,36,37,38,39,40,41,42,43,44,45,46,47,48,49,50,51,52,53] are cited in the Supplementary Materials.
