# Supplementary material for: Transcriptome Analysis Reveals Vimentin-Induced Disruption of Cell–Cell Associations Augments Breast Cancer Cell Migration
Source: Cells. 2022 Dec 13;11(24):4035. doi: 10.3390/cells11244035 (PMC9776984; doi:10.3390/cells11244035)
Supplement: Supplementary file 1 [file cells-11-04035-s001.zip › Supplementary file.pdf]

Article

Transcriptome Analysis Reveals Vimentin-Induced Disruption of Cell–Cell Associations Augments Breast Cancer Cell Migration

Saima Usman [1], Ahmad Jamal [1], Antesar Bushaala [1], Naushin H. Waseem [2], Hebah Al-Dehlawi [3], William Andrew Yeudall[4], Muy-Teck Teh [1], Hemanth Tummala [5] and Ahmad Waseem [1,*]

[1] Centre for Oral Immunobiology and Regenerative Medicine, Institute of Dentistry, Barts and The London School of Medicine and Dentistry, Queen Mary University of London, Newark Street, London E1 2AT, UK

[2] King George V Building, 5th Floor, St Bartholomew's Hospital, Barts NHS Trust, London EC1A 7BE, UK

[3] Department of Oral Diagnostic Sciences, Division of Oral Pathology and Medicine, Faculty of Dentistry, King Abdul Aziz University, Jeddah 21589, Saudi Arabia

[4] Department of Oral Biology and Diagnostic Sciences, The Dental College of Georgia, Augusta University, Augusta, GA 30912, USA

[5] Centre for Genomics and Child Health, Blizard Institute, Barts and The London School of Medicine and Dentistry, Queen Mary University of London, Turner Street, London E1 2AT, UK

* Correspondence: a.waseem@qmul.ac.uk; Tel.: +0044-207-882-2387; Fax: +0044-207-882-7137

Supplementary Data.

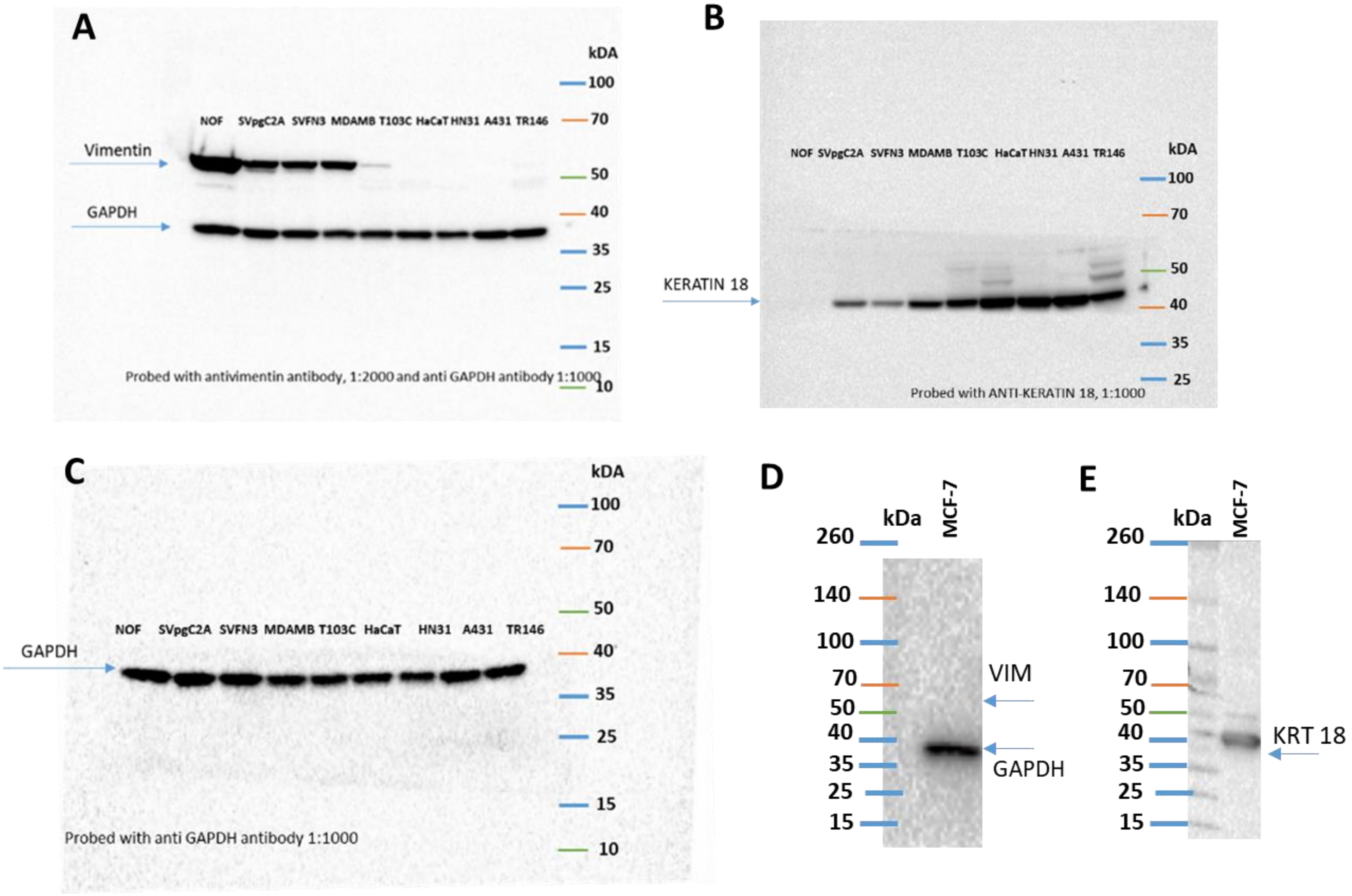

Supplementary Figure S1: **Vimentin and K18 expression in different cell lines.** Ten different cell lines (NOF, SVpgC2A, SVFN3, MDA-MB-231, T103C, HaCaT, HN-31, A431, TR146 (A and B) and MCF-7 (D and E**)** by western blotting. Fifty µg protein was loaded for all the cell lines. GAPDH (C, D) was used as the loading control.

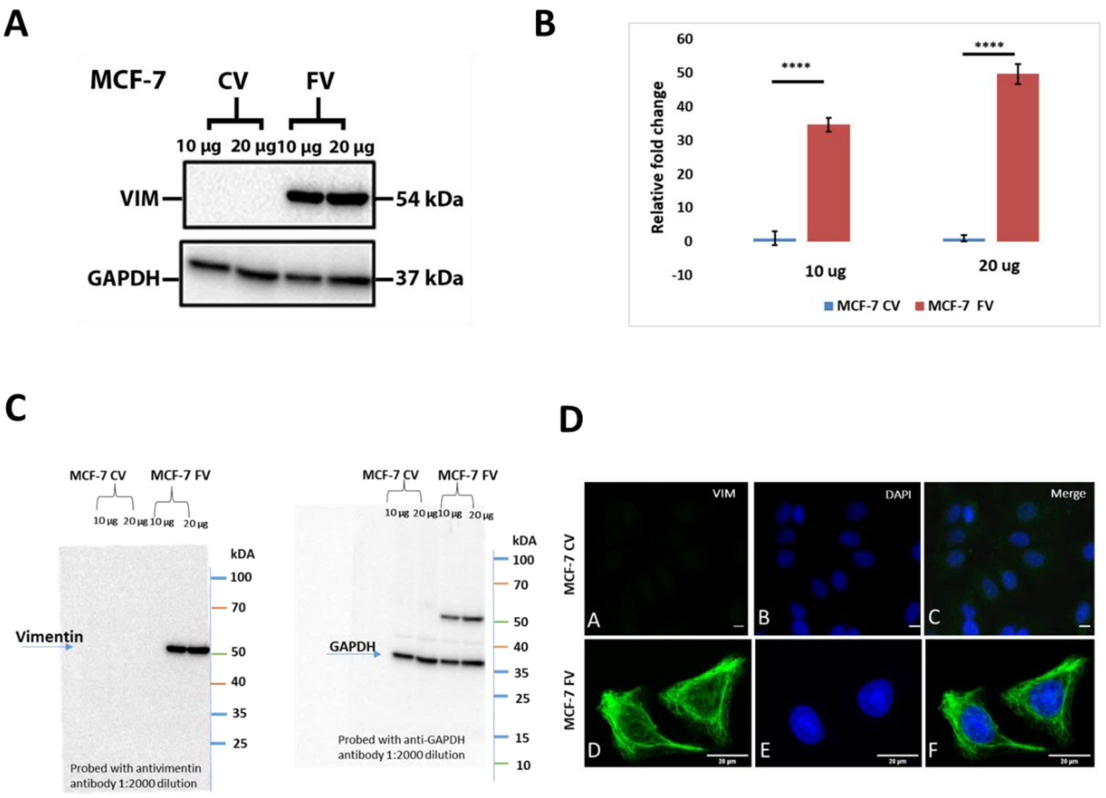

Supplementary Figure S2: **Ectopic expression of vimentin in MCF-7 cells.** (**A)**: MCF-7 cells were transduced with vimentin FV (pLPChygro-VIM) and its vector control CV (pLPChygro) retrovirus. Ten and 20 µg of protein from each transduced cell line was loaded to confirm the transduction efficiency, anti-vimentin antibody was used for probing in 1:2000 dilution. GAPDH was used as the loading control. Relevant bands were cropped from different gels and regrouped. (**B**) Quantification of band intensities using ImageJ software. **(C)** Uncropped original blots. (**D**) Immunostaining in MCF-7 cells transduced with control (pLPC-hygro) CV and (pLPChygro-VIM) FV retroviruses. Cells were immunostained with anti-vimentin primary antibody (abcam ab8069, 1:700 dilution). Alexa Flour 488 (AF 488) labelled goat anti-mouse (Cat # A-11001 Life Technologies, UK, 1:1000 dilution) was used as secondary antibody. Nuclei were stained with DAPI in blue and overlapping images are shown as 'Merge'. Leica DM4000B Epi-fluorescence microscope was used for imaging. (Scale bar = 20 µm). Vimentin filament formation in pLPChygro-VIM expressing MCF-7 cells was seen in green colour (panels D and F) whereas no filament formation in the pLPChygro control (panel A and C) cells. Statistical analyses: n = 3, error bars= ± SEM ****($p<0.0001$).

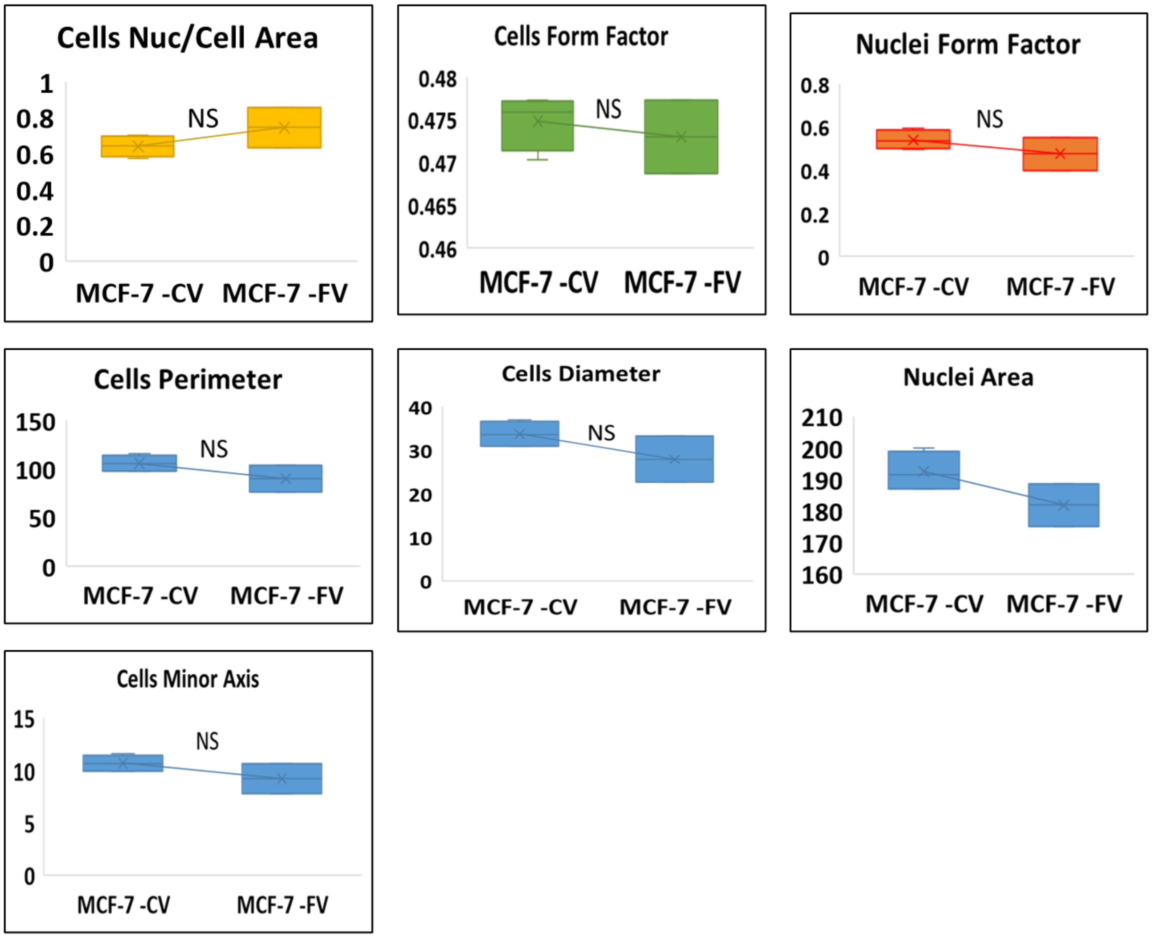

Supplementary Figure S3: Morphology of MCF-7 expressing full-length vimentin. MCF-7 cells expressing pLPChygro-VIM and its vector control pLPChygro were analysed using INCA 2200 analyzer with INCarta software USA. Insignificant data (represented by NS) showing different cell parameters and trends in MCF-7 expressing vimentin. All the cell parameters suggested a decreasing trend while nuclear to cell area is showing increased trends in MCF-7FV. Y-axis shows ratio of area of nucleus to area of cell, cell and nuclei form factor (0-1 and 1=circle), perimeter (µm), cell diameter (µm), nuclear area µm^2, cell minor axis (µm), respectively. Statistical analyses: n = 3, Error bars= ± SEM, Student's t-test was used to calculate p values using Microsoft Excel, NS= not significant

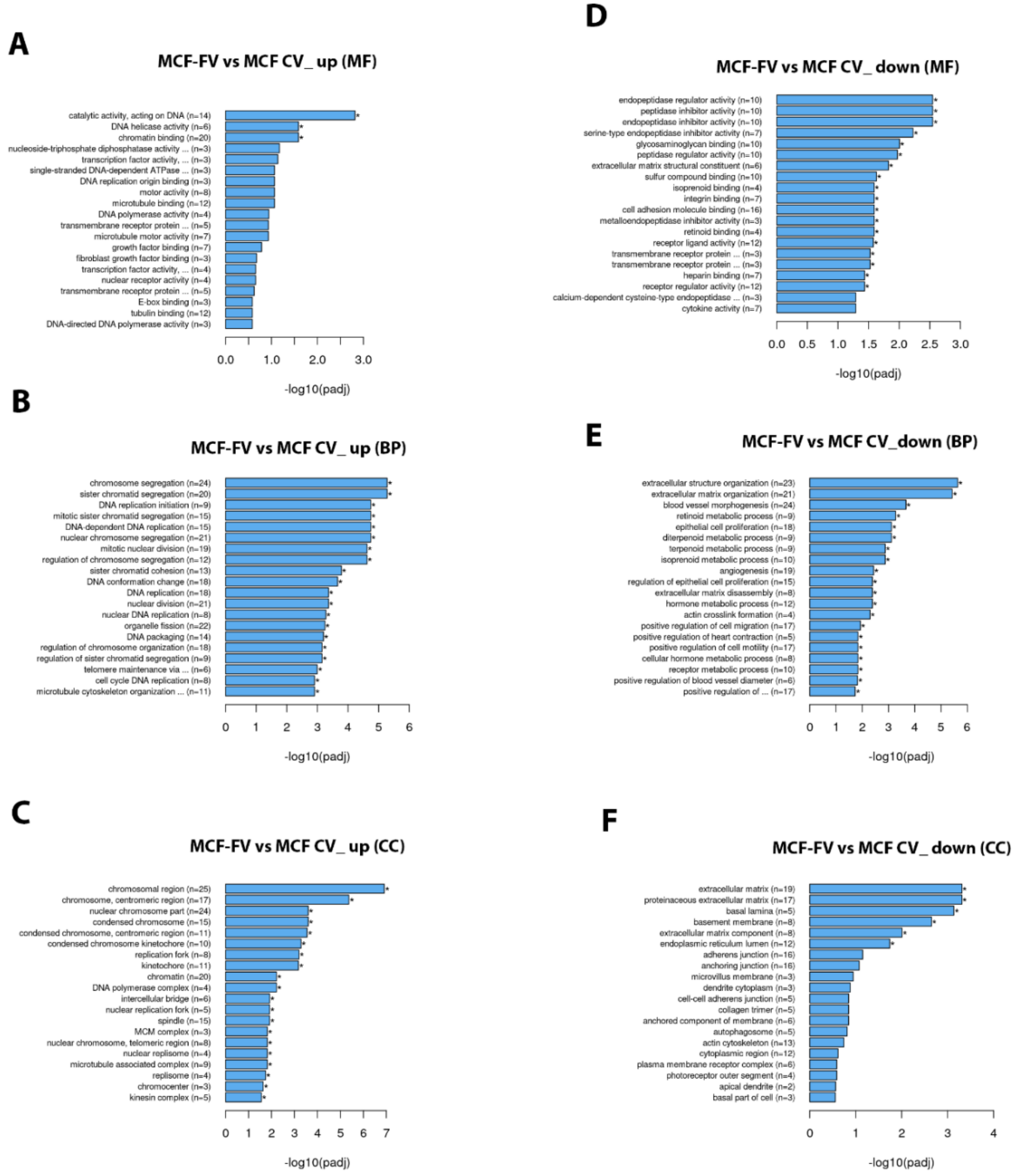

Supplementary Figure S4: GO pathway enrichment analyses of DEGs by RNA-seq. DEGs were grouped into three major functional types: Molecular Function (MF), Biological Process (BP) and Cellular Component (CC). **A**: GO analysis of MF category showing functions that were upregulated by DEGs in MCF-7FV vs MCF-7CV, these include chromatin binding, catalytic activity, acting on DNA and DNA helicase activity. **B**: GO enrichment analysis of BP category

showing biological processes that were upregulated by DEGs, these include chromosome segregation, organelle fission, nuclear chromosome segregation, nuclear division **C**: GO analysis of CC category showing functions that were upregulated by DEGs, these include chromosomal region, nuclear chromosome part, chromatin, chromosome chromosomal centromere region, condensed chromosome, spindle, condensed chromosome, centromeric region **D**: GO analysis of MF category showing functions downregulated by DEGs involved cell adhesion molecule binding, integrin binding, receptor regulator activity and receptor ligand activity **E**: GO analysis of BP category showing processes that were downregulated by DEGs involved blood vessel morphogenesis, extracellular structure organization, extracellular matrix organization, angiogenesis, epithelial cell proliferation, positive regulation of cell migration, positive regulation of cell motility, regulation of epithelial cell proliferation **F**: GO analysis of CC category showing functions downregulated by DEGs, these include extracellular matrix, proteinaceous ECM, basal lamina, basement membrane, ECM components and endoplasmic reticulum lumen, adherens junctions and anchoring junctions.

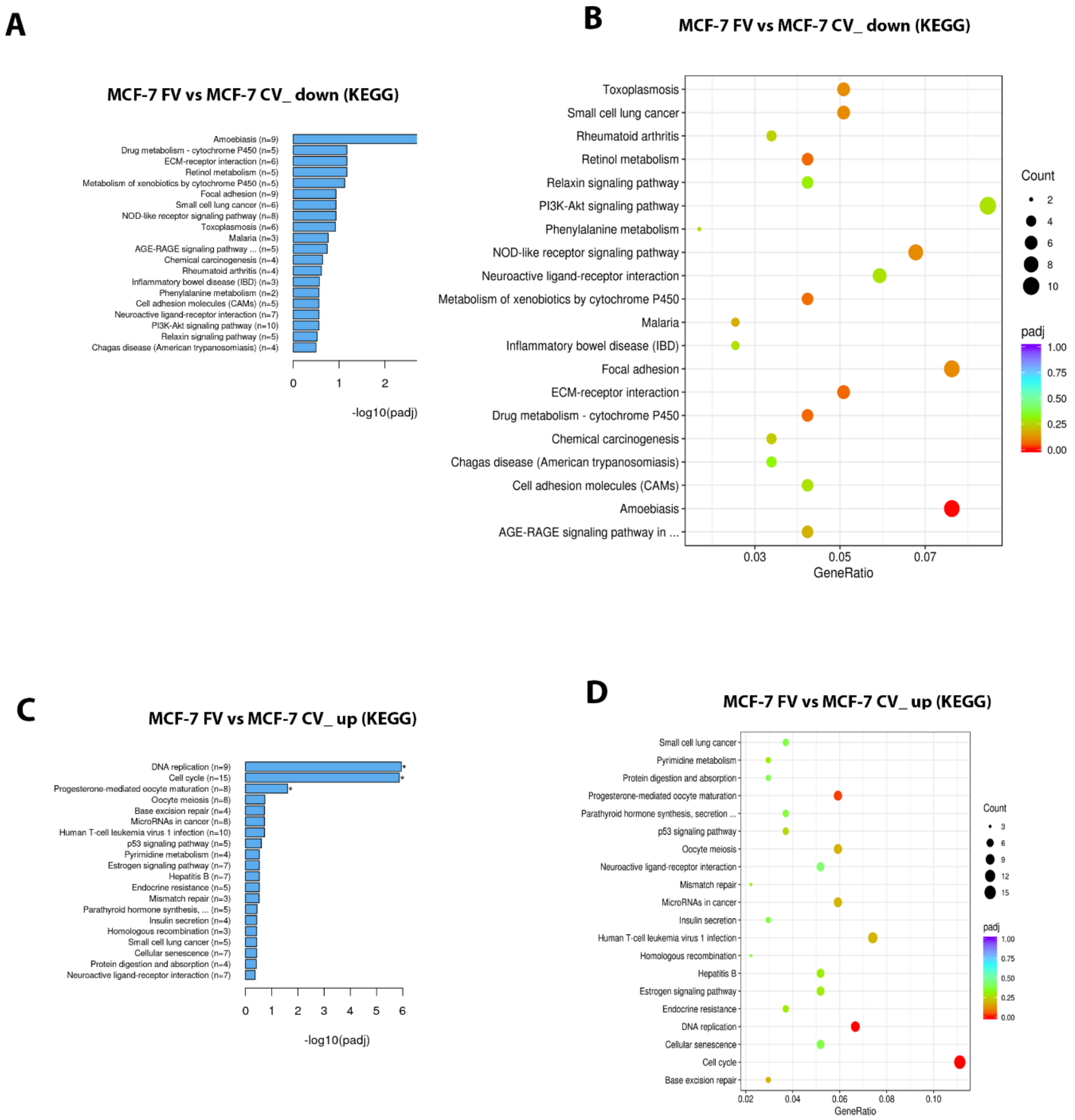

Supplementary Figure S5: KEGG pathway enrichment analyses of DEGs by RNA-Seq. Bar graph **A** and dot plot **B** of downregulated cellular functions involved PI3K-Akt signalling, focal adhesions, ECM-receptor interactions and cell adhesion molecules CAMs. Bar graph **C** and dot plot **D** showing upregulated cellular functions by KEGG analysis in MCF-7 FV vs MCF-7 CV involved cell cycle and DNA replication.

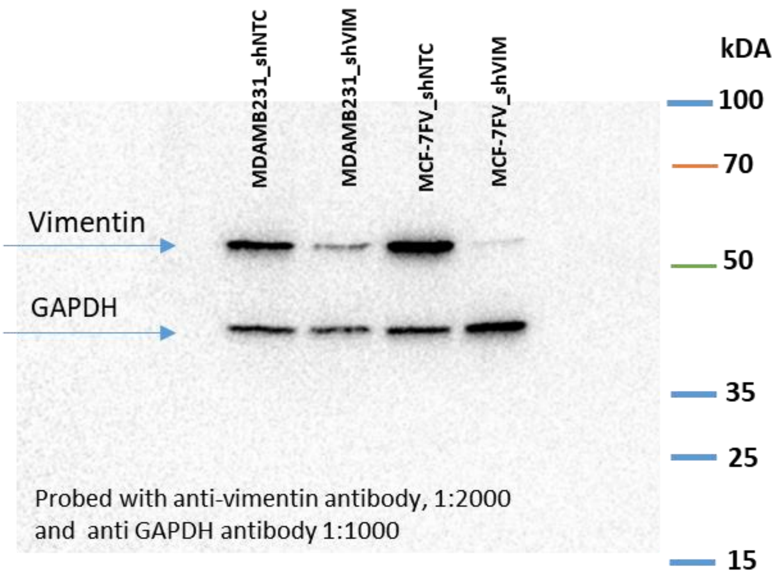

Supplementary Figure S6: Protein expression in MDA-MB-231 and MCF-7-FV cell lines transduced with shVIM and shNTC retroviruses. Cell lines were transduced with VIM sh-RNA and scrambled control NTC retrovirus. A total of 5 µg protein from each transduced cell line was loaded to confirm the transduction efficiency, antivimentin antibody was used for probing in 1:2000 dilution. GAPDH was used as the loading control.

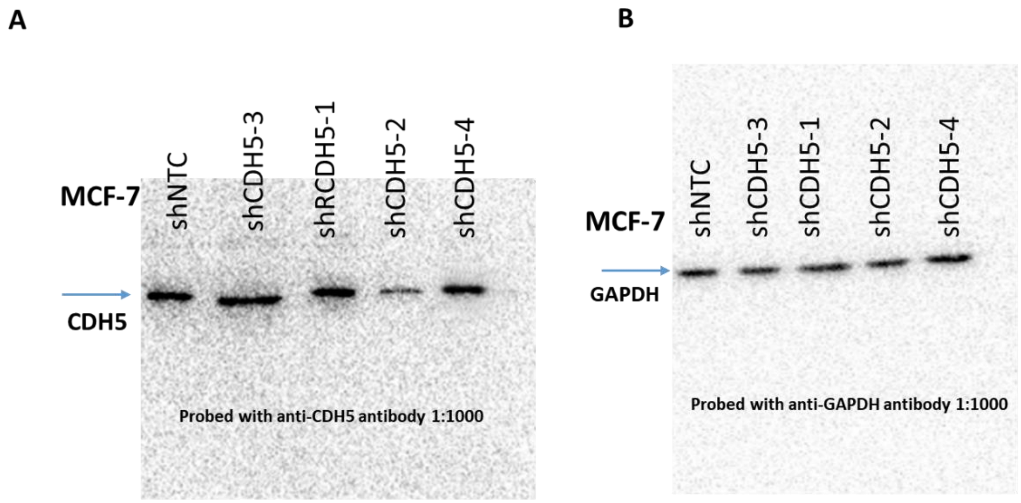

Supplementary Figure S7: CDH5 expression in MCF-7 cell lines transduced with shRNA-CDH5 and shNTC retroviruses. A total of 5 µg protein was loaded for each cell line and probed by western blotting with anti-CDH5 antibody (1:1000) dilution. GAPDH was used as the loading control.

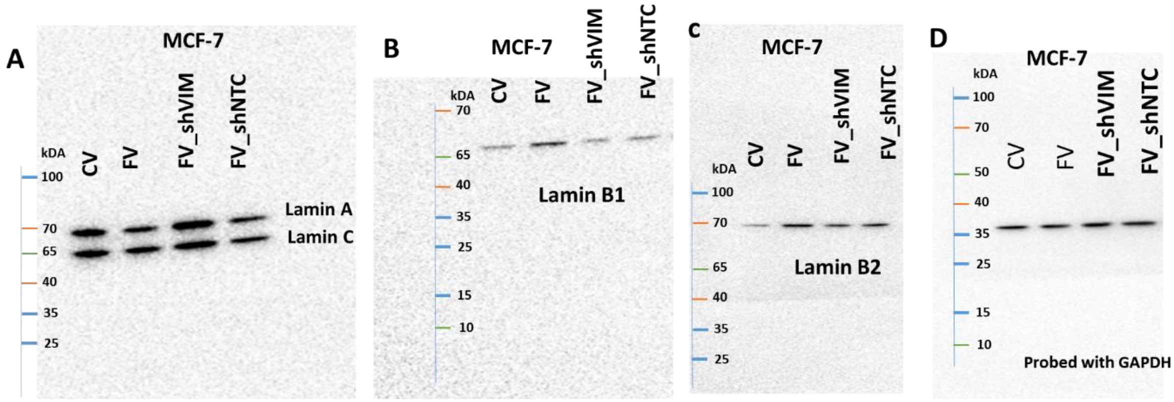

Supplementary Figure S8: Lamin A/C, B1 and B2 expression in MCF-7 cell lines transduced with FV, CV, shVIM and shNTC retroviruses. A total of 10 µg protein was loaded for each cell line and probed with anti-lamin A/C antibody (1:1000) dilution (panel **A**), anti-lamin B1 (1:500) (panel **B**), anti-lamin B2 (1:500) (panel **C**) by western blotting. GAPDH was used as the loading control (panel **D**).

Table S1: Cell lines used in the study.

The cell lines used in this study are listed in the table along with the original reference in which the cell line was generated or first used. These cells were taken from our Institute's liquid nitrogen cell stock. Originally these cells were either bought directly from American Tissue Culture Collection (ATCC) or through Merck UK.

Cell Lines	Origin	Growth Medium and Supplements	Reference
**UK1**	Primary HNSCC derived cell lines	Complete medium*	[32]
**CaLH2**	Primary HNSCC derived cell lines	Complete medium*	[33]
**CaDec12**	Primary HNSCC derived cell lines	Complete medium*	[34]
**SqCC/Y1**	Malignant human buccal squamous cell carcinoma line	Complete medium*	[35]
**SCC4**	Human squamous cell carcinomas (SCCs)	Complete medium*	[36]
**SCC25**	Malignant tongue-derived squamous cell carcinoma keratinocytes	Complete medium*	[36]
**TR146**	Human squamous cell carcinoma	Complete medium*	[37]
**DOK**	Dysplastic or "pre-malignant" oral mucosa	Complete medium*	[38]
**HN6**	Squamous cell carcinoma of the oral tongue	Complete medium*	[39]
**A431**	Epidermoid carcinoma of the vulva	Complete medium*	[40]
**HeLa**	Cervical cancer cells	Complete medium*	[41]
**MDA-MB-231**	Breast adenocarcinoma. Derived from metastatic site: Pleural effusion	Complete medium*	[42]

**MCF-7**	Invasive breast carcinoma of no special type. Derived from metastatic site: Pleural effusion.	Complete medium*	[43]
**SVpgC2a**	Simian virus 40 transformed human premalignant buccal keratinocyte.	Complete medium*	[35]
**SVFN10**	Malignant buccal keratinocyte	Complete medium*	[44]
**SVFN3**	Malignant buccal keratinocyte	Complete medium*	[45]
**HaCaT**	HPV 16-negative, spontaneously immortalized human keratinocyte line	Complete medium*	[46]
**HFF**	Human foreskin fibroblast	Complete medium*	[47]
**NOF**	Normal oral fibroblast	RM+ medium**	[48]
**PDF**	Normal healthy hum-an Dermal Fibroblasts	RM+ medium**	[49]
**H357**	Primary HNSCC derived cell line	RM+ medium**	[34]
**T103C**	HPV 16 immortalized oral keratinocytes	RM+ medium**	[50]
**N/Tert-1**	Epidermal keratinocytes Immortalized by overexpression of hTert and knockdown of p16.	RM+ medium**	[51]
**NEB1**	Immortalized keratinocyte using HPV16 E6/E7	RM+ medium**	[52]
**HN12**	Squamous cell carcinoma of the oral tongue derived from metastatic site: Lymph node	RM+ medium**	[53]

**HN22**	Squamous cell carcinoma of the oral cavity	RM+ medium**	[53]
**HN31**	Head and neck squamous cell carcinoma	RM+ medium**	[53]
**CA1**	Head and neck squamous cell carcinomas (HNSCC)	RM+ medium**	[32]

* DMEM containing 10% v/v foetal calf serum, 50 units/ml penicillin and 50 µg/ml streptomycin.

** Rheinwald Green Modified with Hyclone II: consisting of a 3:1 ratio of DMEM: F12 with 10% Hyclone II serum, to this was added, insulin 5µg/ml, transferrin 5µg/ml, cholera toxin 8.4ng/ml, hydrocortisone 0.4µg/ml, epidermal growth factor 10ng/ml and adenine 24µg/ml. This was designated as RM+ medium.

Table S2: qPCR primers used in this study.

Gene	Product Size (bp)	Primer Sequence (5'-3')	NM #
VIM	123	F> AGGTGGACCAGCTAACCAAC	NM_003380
		R> TTTCGGCTTCCTCTCTCTGA	
YAP1	83	F> CCCAGATGAACGTCACAGC	NM_006106
		R> GATTCTCTGGTTCATGGCTGA	
POLR2A	73	F> GCAAATTCACCAAGAGAGAC	NM_000937
		R> CACGTCGACAGGAACATCAG	
HOXA1	94	F> CAAACATGGATACTTCCTGG	NM_005522
		R> TTTTTGGCTTTTGAAGGGAG	
ADGRF1	197	F> AGGCCATTATGCATTTTCTC	NM_153840
		R> TTATCCCAGACCCCTAAATC	
CDH5	92	F> CGCAATAGACAAGGACATAAC	NM_001795
		R> TATCGTGATTATCCGTGAGG	
AXL	137	F> CATGAAACATGGAGACCTAC	NM_001699
		R> ATCTCTTGGTACTCAGATACTC	
PTPRM	131	F> CCACGAGCTATAAAATTGGAC	NM_001105244
		R> ATCAGCACACTTTGTTCTTG	
CEACAM1	200	F> CCACCTAACAAGATGAATGAAG	NM_001205344
		R> GAATCTCCTAGTGATGAGGG	
TGFBI	146	F> AACCCACAACGAAATCTATG	NM_000660
		R> CTTTTAACTTGAGCCTCAGC	
TFPI2	158	F> CAAGATACAGAACCTGTGATG	NM_006528
		R> AATTTTCCGGATTCTACTGG	
CD109	185	F> AGTATACATATGGGAAGCCAG	NM_001159587
		R> ATCCAGGTATTCAGAAAGTCC	
CDH10	98	F> GGCAGCTAATACAGACTATAAG	NM_006727
		R> TGTGAAGTTTGGATTGACAG	
COL4A3	87	F> AGAAAGCCTATTCCATCAAC	NM_000091
		R> TCAGTGTCTTTTCTTCATGC	
E2F1	133	F> CTGATGAATATCTGTACTACGC	NM_005225
		R> CTTTGATCACCATAACCATCTG	
FSD1	138	F> TTCAGAGCTTTATCTACTCCC	NM_024333
		R> TCATAAGCATGCCTTCTTTC	
BCL2	164	F> GATTGTGGCCTTCTTTGAG	NM_000633
		R> GTTCCACAAAGGCATCC	
CDCA3	189	F> CCAGTTATCTGTTGAGGAAC	NM_031299
		R> TTTGGTTTCCATCTATTGCG	
TUBG1	161	F> GAGAAAAGATCCATGAGGAC	NM_001070
		R> CTTCTTAGGATACCTGTCATTC	
FOXM1	137	F> TCTTTGTTTATCAGTGCTGC	NM_202002
		R> TTTCTTCCTCCTTGATAGTCTG	

CDC20	167	F> CAGCTATATCCTGTCCAGTG	NM_001255
		R> CCAAGTTATCATTACCACCAC	
MATK	172	F> GACATGGTGGAGCATTAC	NM_002378
		R> CTCCAAACTCTCCCTCTC	
CDC45	92	F> GAATTTGCGGGAAATGATTG	NM_001178010
		R> GCTTGAACCCAAAATGAATG	
NES	194	F> ATGGAGACGTCGCTG	NM_006617
		R> ACAGCCAGCTGGAAC	
KIF26A	178	F> CCTCCAAGAGGAAGAAGC	NM_015656
		R> ATAACCTTCACCTTCCCG	
		R> TATAGTCAGATTCCACAGAGG	
LINC00052	198	F> ATGGCAACCATCAAATCAAG	NR_026869
		R> ACACACAGATTGAGAGAGAG	
FGFR4	85	F> CTGAGGACAATGTGATGAAG	NM_002011
		R> CCGTTGCTGGTTTTCTTATAG	
WISP2	155	F> ACACCAATATTAACACGCTG	NM_003881
		R> ATATAGGCTGTGTGTGTAGG	
ITGA2B	178	F> AGAAGAAGGAGAATGAGACC	NM_000419
		R> ATCTTGCTGTTTGGATTCTG	
RAMP3	197	F> GTGTACTATGAGAGTTTCACC	NM_005856
		R> AGAACGACGGGTATAACG	
C15orf59-AS1	160	F> AGATCGATAAGCTAACCTCG	NM_001039614
		R> CTGTCTGAGAGGATATCGG	
KRT8	282	F> AGCTTCTCCGCTCCTTCTAGG	NM_002273
		R> CAGGCTCTGGTTGACCGTAA	
KRT18	117	F> TGATGACACCAATATCACACGA	NM_000224
		R> ATCTGGGCTTGTAGGCCTTT	
KRT19	126	F> GCCACTACTACACGACCATCC	NM_002276
		R> CAAACTTGGTTCGGAAGTCAT	

Table S3: Primers used for cloning shRNAs.

Primer name	Sequence (5'-3')
NTC1_F	GATCTGCTAAGGTTAAGTCGCCCTCTACCTGACCCATAGAGGGCGACTTAACCTTAGTTTTC
NTC1_R	CGAGAAAAACTAAGGTTAAGTCGCCCTCTATGGGTCAGGTAGAGGGCGACTTAACCTTAGCA
NTC2_F	GATCTGCGATAGCGCTAATAATTTTACCTGACCCATAAAATTATTAGCGCTATCGCTTTTC
NTC2_R	TCGAGAAAAAGCGATAGCGCTAATAATTTTATGGGTCAGGTAAAATTATTAGCGCTATCGA
CDH5_shRNA1.F	GATCTGCAAATTCATCTTGAATAATACCTGACCCATATTATTCAAGATGAATTTGCTTTTC
CDH5_shRNA1.R	TCGAGAAAAAGCAAATTCATCTTGAATAATATGGGTCAGGTATTATTCAAGATGAATTTGA
CDH5_shRNA2.F	GATCTGGTATTATCACAATAACGATACCTGACCCATATCGTTATTGTGATAATACCTTTTC
CDH5_shRNA2.R	TCGAGAAAAAGGTATTATCACAATAACGATATGGGTCAGGTATCGTTATTGTGATAATACA
CDH5_shRNA3.F	GATCTGCTCAAAGGAGAATATGTATACCTGACCCATATACATATTCTCCTTTGAGCTTTTC
CDH5_shRNA3.R	TCGAGAAAAAGCTCAAAGGAGAATATGTATATGGGTCAGGTATACATATTCTCCTTTGAGA
CDH5_shRNA4.F	GATCTGCTGGCTACGTATAATAAATACCTGACCCATATTTATTATACGTAGCCAGCTTTTC
CDH5_shRNA4.R	TCGAGAAAAAGAATTCTGGACGTATTATATATGGGTCAGGTATATAATACGTCCAGAATTA

Table S4: Relative *VIM* mRNA fold change expression using qRT-PCR in 28 different cell lines normalized to PDF.

Cell Lines	% Fold Change Relative to PDF	Genes	±SEM
**PDF**	100	*VIM*	2.920806109
**NOF**	74.16819492	*VIM*	3.8964665
**HFF**	66.30861933	*VIM*	12.20810502
**SVFN10**	44.44153349	*VIM*	4.748625576
**SVpgC2a**	36.69548511	*VIM*	0.568106051
**SVFN3**	14.29045498	*VIM*	1.185612628
**HeLa**	9.000960615	*VIM*	0.04507798
**MDAMB-231**	3.356038774	*VIM*	0.983687977
**CaDec12**	3.19710069	*VIM*	0.075953309
**NEB1**	3.123744651	*VIM*	0.188339506
**SCC25**	3.049515326	*VIM*	0.02346525
**HN6**	2.673128984	*VIM*	0.294550637
**T103C**	2.335167234	*VIM*	0.212422262
**CA1**	2.129944983	*VIM*	0.014202651
**SCC4**	1.497685792	*VIM*	0.042978458
**H357**	1.266963584	*VIM*	0.126835851
**N/Tert-1**	0.946205572	*VIM*	0.402552537
**TR146**	0.897825517	*VIM*	0.066381957
**HN22**	0.849969435	*VIM*	0.065517448
**HN12**	0.736966204	*VIM*	0.015869919
**DOK**	0.532005938	*VIM*	0.022724242
**A431**	0.429918784	*VIM*	0.046498245
**UK1**	0.278752947	*VIM*	0.000370504
**MCF7**	0.171251419	*VIM*	0.017475436
**SqCC/Y1**	0.086455331	*VIM*	0.048412516
**CaLH2**	0.063313248	*VIM*	0.004631299
**HaCaT**	0.047070125	*VIM*	0.016363924
**HN31**	0.036328705	*VIM*	0.002964032

Table S5: Relative *KRT18* mRNA fold change expression using qRT-PCR in 28 different cell lines normalized to MCF-7.

Cell Lines	% Fold Change relative to MCF-7	Gene	±SEM
**MCF7**	100	*KRT18*	1.572675555
**HeLa**	85.91638514	*KRT18*	0.373261603
**A431**	35.28997748	*KRT18*	2.488410688
**HN6**	25.67567568	*KRT18*	6.698801573
**H357**	22.16356982	*KRT18*	0.373261603
**HN31**	22.00872748	*KRT 18*	0.054745035
**HN12**	18.55996622	*KRT18*	2.901486863
**CaDec12**	13.26858108	*KRT18*	0.134374177
**CaLH2**	13.08136261	*KRT18*	0.389187432
**HaCaT**	10.77280405	*KRT18*	0.843073541
**MDAMB-231**	9.252533784	*KRT18*	0.285669547
**TR146**	9.181447072	*KRT18*	0.145820866
**SqCC/Y1**	6.50267455	*KRT18*	0.066191724
**DOK**	6.084600225	*KRT18*	0.878408973
**SCC4**	4.755067568	*KRT18*	0.147313913
**CA1**	4.47142455	*KRT18*	0.164732788
**N/Tert-1**	3.777449324	*KRT18*	1.437803696
**HN22**	3.517736486	*KRT18*	0.056735764
**T103C**	3.252393018	*KRT18*	0.038321525
**HFF**	3.158783784	*KRT18*	0.233910605
**SVpgC2a**	2.134712838	*KRT18*	0.224454644
**UK1**	2.117117117	*KRT18*	0.026874835
**SVFN10**	1.538780968	*KRT18*	0.253668586
**NEB1**	1.37915259	*KRT18*	0.306821038
**SVFN3**	0.761542793	*KRT18*	0.003384239
**SCC25**	0.568552928	*KRT18*	0.095754043
**PDF**	0.489864865	*KRT18*	0.16771888
**NOF**	0.05567286	*KRT18*	0.023639902

Table S6: Log2-Fold Change in expression of different keratins and lamins in MCF-7FV vs MCF-7CV.

Gene_id	Genes	log2-Fold Change	padj
ENSG00000170477	*KRT4*	-4.275879737	1.02E-07
ENSG00000128422	*KRT17*	-1.53434516	0.184684652
ENSG00000167767	*KRT80*	-0.895784691	0.420669306
ENSG00000171345	*KRT19*	0.765372084	0.562294166
ENSG00000170421	*KRT8*	0.585696454	0.693118364
ENSG00000111057	*KRT18*	0.462540454	0.774427901
ENSG00000186395	*KRT10*	-0.3001551	0.859794897
ENSG00000135480	*KRT7*	0.270910074	0.896277816
ENSG00000171346	*KRT15*	0.259630945	0.898777835
ENSG00000171403	*KRT9*	0.79870705	1
ENSG00000186081	*KRT5*	0.603948173	1
ENSG00000205426	*KRT81*	-0.103897604	0.963813058
ENSG00000186847	*KRT14*	0.34051462	1
ENSG00000161849	*KRT84*	0.306662331	1
ENSG00000186832	*KRT16*	-0.034970388	0.992118516
ENSG00000135443	*KRT85*	-0.033899175	0.995213102
ENSG00000113368	*LMNB1*	1.438271219	0.05817484
ENSG00000176619	*LMNB2*	1.437057843	0.120994885
ENSG00000160789	*LMNA*	1.070266529	0.396107944

Table S7: List of upregulated DEGs by RNA-Seq analysis (log2-Fold Change >3.01).

Gene name	Gene id	MCF7_ FV1	MCF7_ FV2	MCF7_CV1	MCF7_CV2	log2-Fold Change	P value	P adj
VIM	ENSG00000026025	20750.97	25241.69	13.36005	12.59617	10.7916	6.29E-85	1.14E-80
C15orf59-AS1	ENSG00000260469	103.0999	71.58784	0	0	8.999666	2.62E-08	1.01E-05
-	novel.586	14.55527	803.2713	2.862868	0.899726	7.76841	0.000384	0.019064
RN7SL674P	ENSG00000239899	21.83291	32.53993	0	0	7.320044	4.60E-05	0.003957
ASCL1	ENSG00000139352	139.488	127.3706	1.908578	0	7.162902	3.02E-09	1.53E-06
RAMP3	ENSG00000122679	172.2374	51.13417	0	1.799453	6.919595	7.07E-08	2.18E-05
KLK11	ENSG00000167757	25.47173	12.08626	0	0	6.778762	0.000402	0.01967
SOAT2	ENSG00000167780	42.45288	31.61021	0	0.899726	6.322181	0.000211	0.012566
AC098935.1	ENSG00000226945	75.20224	62.29071	0	1.799453	6.221677	1.13E-06	0.00023
MFAP2	ENSG00000117122	65.49873	28.82108	0.954289	0.899726	5.665261	2.45E-05	0.00244
-	novel.455	3.638818	72.51755	1.908578	0	5.350689	0.00065	0.027506
SMTNL2	ENSG00000188176	88.54458	39.97762	1.908578	1.799453	5.112103	1.81E-06	0.000326
GPR68	ENSG00000119714	2340.973	862.7729	54.39448	40.48768	5.077783	2.25E-17	1.02E-13
C15orf59	ENSG00000205363	2419.814	1057.083	56.30306	48.58522	5.050895	6.22E-19	5.65E-15
BRD2	ENSG00000204256	4.851758	79.02553	0.954289	1.799453	4.928887	0.000394	0.019361
AC055854.1	ENSG00000253125	154.0433	71.58784	5.725735	1.799453	4.914738	2.03E-08	8.03E-06
RN7SL4P	ENSG00000263740	100.674	2.789136	0.954289	2.699179	4.80898	0.000427	0.020458
RPS2P7	ENSG00000235508	167.3856	13.94568	3.817157	2.699179	4.796914	9.95E-06	0.001255
AC083967.1	ENSG00000254337	71.56343	95.76035	3.817157	2.699179	4.687931	1.27E-07	3.35E-05
MAT1A	ENSG00000151224	26.68467	20.45367	0.954289	0.899726	4.666936	0.001488	0.046748
CYP4F22	ENSG00000171954	309.2996	207.3258	14.31434	7.19781	4.589825	3.39E-12	4.72E-09
PYCR3	ENSG00000104524	661.052	105.0575	17.17721	17.0948	4.480973	6.43E-09	3.07E-06

BRINP2	ENSG00000198797	612.5344	389.5494	27.67439	18.89425	4.428632	2.19E-14	4.98E-11
AL135818.2	ENSG00000260810	144.3398	123.6517	2.862868	9.896989	4.382329	1.05E-08	4.67E-06
SYNDIG1	ENSG00000101463	52.15639	25.10223	1.908578	1.799453	4.377086	0.000152	0.010005
**-**	novel.221	214.6903	196.1693	8.588603	12.59617	4.27439	4.66E-11	5.29E-08
PGR	ENSG00000082175	477.8981	630.3448	33.40012	26.09206	4.220836	1.56E-14	4.05E-11
**-**	novel.45	120.081	104.1278	8.588603	3.598905	4.208184	5.27E-08	1.77E-05
PCDH10	ENSG00000138650	1949.194	1028.262	97.3375	71.9781	4.136454	2.84E-14	5.74E-11
LINC01016	ENSG00000249346	88.54458	72.51755	5.725735	3.598905	4.113893	8.45E-07	0.000186
AL135818.1	ENSG00000258875	35.17524	28.82108	1.908578	1.799453	4.108482	0.00048	0.021955
S1PR5	ENSG00000180739	50.94346	27.89136	0	4.498631	4.107791	0.000445	0.020902
ACTL8	ENSG00000117148	86.1187	39.97762	2.862868	4.498631	4.090067	1.25E-05	0.001503
CXCL12	ENSG00000107562	3398.656	3394.379	199.4464	199.7392	4.088921	7.73E-18	4.68E-14
PGLYRP2	ENSG00000161031	168.5986	48.34503	5.725735	7.19781	4.063703	1.36E-06	0.000265
**-**	novel.476	681.672	495.5366	24.81152	46.78577	4.036927	1.31E-12	2.38E-09
GPER1	ENSG00000164850	2624.801	704.7218	125.9662	81.87509	4.00179	4.13E-10	3.41E-07
GREB1	ENSG00000196208	23290.86	14261.78	1307.376	1127.357	3.947074	2.13E-15	6.45E-12
ITGA2B	ENSG00000005961	209.8385	98.54949	10.49718	9.896989	3.916536	3.81E-08	1.36E-05
**-**	novel.472	420.89	12.08626	15.26863	13.49589	3.910292	0.000103	0.007591
HEY2	ENSG00000135547	414.8253	242.6549	14.31434	29.69097	3.897238	5.01E-10	3.96E-07
ASTL	ENSG00000188886	298.3831	171.067	20.99436	15.29535	3.69352	4.88E-09	2.40E-06
AC079140.2	ENSG00000250321	55.79521	16.73482	3.817157	1.799453	3.690815	0.001066	0.038274
**-**	novel.674	551.8874	26.03194	25.76581	19.79398	3.663921	7.80E-05	0.00603
KIAA1143	ENSG00000163807	3.638818	170.1373	4.771446	8.997263	3.65919	0.001313	0.043267
KCNF1	ENSG00000162975	408.7606	136.6677	18.13149	25.19234	3.651066	7.32E-08	2.21E-05
C5AR2	ENSG00000134830	1212.939	752.1371	82.06887	80.07564	3.59889	6.69E-12	8.10E-09
PEG10	ENSG00000242265	816.3082	1067.31	71.57169	85.474	3.584149	2.35E-12	3.56E-09
PLIN5	ENSG00000214456	295.9572	134.8083	21.94865	14.39562	3.567136	7.77E-08	2.28E-05

UBE2SP2	ENSG00000224126	659.839	92.97122	41.03444	22.49316	3.566548	5.23E-06	0.000743
RBM24	ENSG00000112183	272.9114	272.4057	18.13149	27.89152	3.564703	1.44E-09	8.17E-07
-	novel.507	195.2832	142.246	13.36005	15.29535	3.556209	3.60E-08	1.31E-05
SIM1	ENSG00000112246	36.38818	37.18849	1.908578	4.498631	3.514053	0.000739	0.030302
GGT6	ENSG00000167741	218.3291	79.95525	16.22292	9.896989	3.513173	1.75E-06	0.000321
TEX14	ENSG00000121101	326.2807	354.2203	38.17157	23.39288	3.468398	1.34E-09	8.17E-07
MSRA	ENSG00000175806	104.3128	26.96165	6.680024	6.298084	3.333328	0.000256	0.01445
DUSP9	ENSG00000130829	242.5879	72.51755	16.22292	16.19507	3.277966	7.94E-06	0.001045
WISP2	ENSG00000064205	17719.83	5241.717	1255.845	1146.251	3.256803	5.40E-08	1.78E-05
GNG13	ENSG00000127588	265.6337	79.95525	16.22292	20.6937	3.222983	8.19E-06	0.001055
GABRD	ENSG00000187730	261.9949	69.72841	17.17721	18.89425	3.197713	1.58E-05	0.001766
CT62	ENSG00000225362	63.07285	63.22043	5.725735	8.097537	3.189058	9.86E-05	0.007333
RPS2P55	ENSG00000216866	793.2624	91.11179	58.21164	40.48768	3.162743	6.06E-05	0.00497
AP002884.1	ENSG00000250303	172.2374	85.53352	16.22292	12.59617	3.159722	6.49E-06	0.000887
FAM156A	ENSG00000268350	152.8304	22.31309	14.31434	5.398358	3.15066	0.000756	0.030633
METTL7A	ENSG00000185432	654.9873	857.1946	85.88603	86.37372	3.134261	8.56E-10	5.75E-07
SUSD3	ENSG00000157303	1063.748	330.9775	83.02316	76.47674	3.127605	4.96E-07	0.000114
NMU	ENSG00000109255	78.84106	72.51755	5.725735	11.69644	3.113991	6.98E-05	0.005513
MALRD1	ENSG00000204740	234.0973	235.2172	24.81152	29.69097	3.105442	8.88E-08	2.48E-05
BRINP3	ENSG00000162670	259.569	388.6197	44.85159	30.59069	3.104963	7.42E-08	2.21E-05
ANPEP	ENSG00000166825	84.90576	39.04791	9.542892	5.398358	3.051944	0.000372	0.018743
CTIF	ENSG00000134030	154.0433	66.00956	8.588603	17.99453	3.042788	5.69E-05	0.004738
TUBBP2	ENSG00000214222	4962.135	639.642	374.0814	315.8039	3.021268	3.56E-05	0.003247
-	novel.224	500.944	401.6357	59.16593	52.18413	3.018873	1.39E-08	5.63E-06
PHRF1	ENSG00000070047	214.6903	17.66453	12.40576	16.19507	3.017531	0.001202	0.040953
NPY1R	ENSG00000164128	890.2975	2574.373	204.2179	224.9316	3.013461	2.74E-07	6.64E-05
SLC7A8	ENSG00000092068	392.9924	201.7475	46.76017	26.99179	3.011697	1.02E-06	0.000215

Table S8: List of downregulated DEGs by RNA-Seq analysis (log2-Fold Change range from −6 to −3).

Gene	Gene id	MCF7_ FV1	MCF7_ FV2	MCF7_ CV1	MCF7_ CV2	log2 Fold Change	p value	p adj
LINC02263	ENSG00000228358	0	0	17.17721	25.19234	-6.76145	0.000283	0.015711
SAMD9L	ENSG00000177409	0	0	11.45147	24.29261	-6.51658	0.000737	0.03029
LINC01484	ENSG00000253686	0	0	25.76581	9.896989	-6.51115	0.000794	0.031984
-	novel.676	0	0.929712	85.88603	0.899726	-6.37769	0.000823	0.032621
RGS7BP	ENSG00000186479	0	0.929712	33.40012	35.98905	-6.04882	0.000332	0.017153
HOXA1	ENSG00000105991	1.212939	0.929712	41.98872	43.18686	-5.33197	6.29E-05	0.005079
ITIH2	ENSG00000151655	0	1.859424	41.03444	35.98905	-5.23903	0.000148	0.009848
MYO3B	ENSG00000071909	0	1.859424	14.31434	52.18413	-5.03805	0.000624	0.026625
ADGRF1	ENSG00000153292	1.212939	0.929712	24.81152	37.7885	-4.88709	0.000483	0.021963
CDH5	ENSG00000179776	15.76821	13.01597	437.0644	352.6927	-4.7845	1.57E-15	5.70E-12
-	novel.432	1.212939	0.929712	41.98872	16.19507	-4.77914	0.000918	0.034805
TLL1	ENSG00000038295	1.212939	1.859424	34.35441	49.48495	-4.74428	8.18E-05	0.006286
LINC00284	ENSG00000233725	7.277637	13.01597	245.2523	260.9206	-4.61886	1.73E-12	2.86E-09
LIPG	ENSG00000101670	4.851758	2.789136	89.70318	93.57154	-4.61372	1.16E-07	3.09E-05
AC005821.1	ENSG00000265702	0	3.718849	32.44583	45.88604	-4.2924	0.000317	0.016626
KRT4	ENSG00000170477	24.25879	7.437697	313.0069	288.8121	-4.27588	1.02E-10	1.02E-07
LONRF3	ENSG00000175556	1.212939	1.859424	26.7201	28.79124	-4.15134	0.001306	0.043126
RASGRP3	ENSG00000152689	2.425879	6.507985	69.66311	92.67181	-4.14165	1.87E-06	0.000329
PDZK1IP1	ENSG00000162366	4.851758	0	39.12586	35.98905	-4.05515	0.000684	0.028476
A2M	ENSG00000175899	1.212939	5.578273	69.66311	47.68549	-4.05035	3.37E-05	0.003153
HPGD	ENSG00000164120	8.490576	13.94568	150.7777	187.1431	-3.89645	8.14E-09	3.69E-06
SLC26A7	ENSG00000147606	0	4.648561	31.49154	42.28714	-3.89616	0.000866	0.033603
PTPRM	ENSG00000173482	48.51758	27.89136	560.1678	544.3344	-3.86252	3.64E-12	4.72E-09
AL691482.3	ENSG00000249007	4.851758	13.01597	146.0062	112.4658	-3.82271	2.48E-07	6.16E-05
AC006372.1	ENSG00000223872	3.638818	4.648561	44.85159	70.17865	-3.78435	4.47E-05	0.003881
STEAP4	ENSG00000127954	3.638818	10.22683	86.84032	100.7693	-3.72245	3.04E-06	0.000482
SLC12A1	ENSG00000074803	0	5.578273	43.8973	34.1896	-3.72171	0.001015	0.037227
LINC00987	ENSG00000237248	1.212939	14.87539	121.1947	96.27071	-3.70616	1.25E-05	0.001503
LINC00930	ENSG00000258647	4.851758	2.789136	31.49154	65.68002	-3.69378	0.000208	0.012474
TNFSF15	ENSG00000181634	12.12939	29.75079	237.618	302.308	-3.67001	7.56E-09	3.52E-06
ANKRD22	ENSG00000152766	8.490576	35.32906	227.1208	301.4083	-3.57002	2.00E-07	5.05E-05
CP	ENSG00000047457	29.11055	75.30668	627.9223	578.524	-3.52056	2.17E-09	1.13E-06
STAT4	ENSG00000138378	1.212939	14.87539	101.1547	88.17318	-3.5078	5.00E-05	0.004245
AL133370.1	ENSG00000258837	1.212939	12.08626	67.75453	89.0729	-3.50757	8.30E-05	0.006335

LINC01768	ENSG00000228420	2.425879	4.648561	28.62868	53.08385	-3.50348	0.000683	0.028476
ABCA4	ENSG00000198691	38.81406	39.04791	392.2129	475.0555	-3.47745	1.61E-10	1.46E-07
AASS	ENSG00000008311	25.47173	43.69647	361.6756	403.9771	-3.45952	1.36E-09	8.17E-07
AC010998.3	ENSG00000278484	1.212939	7.437697	60.12022	38.68823	-3.45822	0.000487	0.022059
SYT16	ENSG00000139973	3.638818	5.578273	49.62304	51.2844	-3.4349	0.000201	0.012354
-	novel.138	16.98115	19.52396	183.2235	212.3354	-3.43413	2.85E-08	1.08E-05
SERPINA12	ENSG00000165953	2.425879	3.718849	27.67439	39.58796	-3.43395	0.001541	0.047679
TCIM	ENSG00000176907	19.40703	67.86899	412.2529	528.1393	-3.41723	8.31E-08	2.39E-05
HOTAIRM1	ENSG00000233429	9.703515	16.73482	138.3719	143.9562	-3.401	5.18E-07	0.000118
CYP2B7P	ENSG00000256612	3.638818	4.648561	41.03444	46.78577	-3.39527	0.000443	0.020822
LGR6	ENSG00000133067	8.490576	0.929712	41.03444	52.18413	-3.36785	0.000965	0.036349
FYB1	ENSG00000082074	1.212939	7.437697	38.17157	53.08385	-3.34528	0.000855	0.033362
TMEM51-AS1	ENSG00000175147	2.425879	8.367409	63.93738	46.78577	-3.31787	0.000298	0.016101
CEACAM6	ENSG00000086548	72.77637	155.2619	1102.204	1166.945	-3.31045	1.41E-09	8.17E-07
AC004925.1	ENSG00000279419	1.212939	6.507985	34.35441	44.08659	-3.29183	0.001589	0.04866
GSTM2	ENSG00000213366	15.76821	14.87539	148.8691	147.5551	-3.27565	4.67E-07	0.000109
CGA	ENSG00000135346	16.98115	13.01597	165.092	123.2625	-3.27213	8.51E-07	0.000186
AL121790.2	ENSG00000259087	10.91645	2.789136	73.48027	54.8833	-3.2683	0.000212	0.012603
-	novel.2	36.38818	19.52396	271.9724	260.0209	-3.26185	5.52E-08	1.79E-05
SHROOM1	ENSG00000164403	117.6551	38.1182	728.1227	719.781	-3.22385	1.00E-07	2.74E-05
CREB3L1	ENSG00000157613	40.027	21.38338	281.5153	285.2132	-3.21712	6.14E-08	1.96E-05
ABCC4	ENSG00000125257	109.1645	141.3162	1118.427	1197.536	-3.20716	1.28E-10	1.23E-07
A2MP1	ENSG00000256069	46.0917	61.361	498.139	488.5514	-3.19519	2.03E-09	1.12E-06
CXCL8	ENSG00000169429	20.61997	112.4952	496.2304	719.781	-3.18264	4.38E-06	0.000658
VTCN1	ENSG00000134258	116.4422	121.7923	1086.935	1004.994	-3.13405	2.24E-10	1.94E-07
CARD6	ENSG00000132357	2.425879	8.367409	40.08015	56.68276	-3.12592	0.000929	0.035062
DNM3	ENSG00000197959	12.12939	24.17252	130.7376	188.9425	-3.12347	2.87E-06	0.000466
PRKG1	ENSG00000185532	40.027	80.88496	508.6361	534.4374	-3.10174	3.50E-08	1.30E-05
CEACAM1	ENSG00000079385	10.91645	9.297122	71.57169	97.17044	-3.06682	4.60E-05	0.003957
EDIL3	ENSG00000164176	27.89761	61.361	368.3556	382.3837	-3.06262	1.81E-07	4.64E-05
RGS2	ENSG00000116741	7.277637	22.31309	116.4233	134.9589	-3.06249	2.15E-05	0.002215
AC091544.4	ENSG00000260337	13.34233	26.03194	143.1434	187.1431	-3.05424	3.05E-06	0.000482
BCAS1	ENSG00000064787	2921.971	3150.794	23739.85	26611.2	-3.05157	6.20E-11	6.62E-08
EDN1	ENSG00000078401	151.6174	238.936	1489.645	1726.575	-3.03994	2.17E-09	1.13E-06
SLC4A4	ENSG00000080493	12.12939	13.94568	86.84032	126.8614	-3.03126	1.76E-05	0.001904
FOLH1	ENSG00000086205	26.68467	26.96165	201.355	234.8286	-3.02326	3.08E-07	7.35E-05
AC091544.2	ENSG00000258676	10.91645	13.94568	114.5147	86.37372	-3.00708	2.50E-05	0.002484

Table S9: **List of upregulated long non-coding RNAs using RNA-Seq analysis (log2-Fold Change >1.7).**

Gene	Gene_id	MCF7_ FV1	MCF7_ FV2	MCF7_ CV1	MCF7_ CV2	log2-Fold Change	p value	p adj
C15orf59-AS1	ENSG00000260469	103.0999	71.58784	0	0	8.999666	2.62E-08	1.01E-05
AC083967.1	ENSG00000254337	71.56343	95.76035	3.817157	2.699179	4.687931	1.27E-07	3.35E-05
AL135818.2	ENSG00000260810	144.3398	123.6517	2.862868	9.896989	4.382329	1.05E-08	4.67E-06
LINC01016	ENSG00000249346	88.54458	72.51755	5.725735	3.598905	4.113893	8.45E-07	0.000186
AP002884.1	ENSG00000250303	172.2374	85.53352	16.22292	12.59617	3.159722	6.49E-06	0.000887
LINC02568	ENSG00000259459	53.36933	81.81467	6.680024	11.69644	2.878053	0.00032	0.016727
LINC00052	ENSG00000259527	208.6256	398.8465	45.80588	44.08659	2.757977	2.68E-06	0.000442
LINC00992	ENSG00000248663	411.1865	341.2044	125.0119	94.47126	1.77735	0.000788	0.031799
AC074135.1	ENSG00000267886	366.3077	256.6006	102.1089	82.77482	1.751876	0.001322	0.043505
SNHG15	ENSG00000232956	1508.897	831.1627	371.2185	331.999	1.73416	0.000913	0.034744

Table S10: List of downregulated long non-coding RNAs using RNA-Seq analysis (log2-Fold Change range from −6 to 0.0008).

Gene	Gene id	MCF7_ FV1	MCF7_ FV2	MCF7_ CV1	MCF7_ CV2	log2-Fold Change	p value	p adj
LINC02263	ENSG00000228358	0	0	17.17721	25.19234	-6.76145	0.000283	0.015711
LINC01484	ENSG00000253686	0	0	25.76581	9.896989	-6.51115	0.000794	0.031984
LINC00284	ENSG00000233725	7.277637	13.01597	245.2523	260.9206	-4.61886	1.73E-12	2.86E-09
AC006372.1	ENSG00000223872	3.638818	4.648561	44.85159	70.17865	-3.78435	4.47E-05	0.003881
LINC00987	ENSG00000237248	1.212939	14.87539	121.1947	96.27071	-3.70616	1.25E-05	0.001503
LINC00930	ENSG00000258647	4.851758	2.789136	31.49154	65.68002	-3.69378	0.000208	0.012474
LINC01768	ENSG00000228420	2.425879	4.648561	28.62868	53.08385	-3.50348	0.000683	0.028476
AC010998.3	ENSG00000278484	1.212939	7.437697	60.12022	38.68823	-3.45822	0.000487	0.022059
AL121790.2	ENSG00000259087	10.91645	2.789136	73.48027	54.8833	-3.2683	0.000212	0.012603
LINC02015	ENSG00000231574	81.26694	84.60381	608.8365	629.8084	-2.90026	1.27E-08	5.35E-06
LINC01164	ENSG00000189275	18.19409	11.15655	104.9718	96.27071	-2.7899	7.37E-05	0.005769
AL590004.4	ENSG00000260604	99.46103	153.4025	753.8885	924.0189	-2.7277	1.61E-07	4.17E-05
UCA1	ENSG00000214049	41.23994	39.04791	253.8409	233.9288	-2.60374	4.16E-06	0.00063
NEAT1	ENSG00000245532	4901.488	7187.605	29914.1	31052.25	-2.33425	1.34E-06	0.000264
AC015712.1	ENSG00000232386	93.39634	127.3706	420.8415	425.5705	-1.93675	0.000223	0.01304
ERVE-1	ENSG00000267259	43.66582	73.44726	214.7151	233.9288	-1.93248	0.000858	0.033362

Table S11: Comparison of relative log2-Fold Changes in DEGs of interest between MCF-7FV and MCF-7FV_ shVIM. The relative log2-fold changes in DEGs between MCF-7FV and MCF-7FV_ shVIM was calculated to determine "reversibility" in their expression after vimentin knockdown by shVIM RNA. The gene expression was considered "reversed" if positive log2-fold values in MCF-7FV became negative or going towards negative in MCF-7FV_sh-VIM and vice versa. Except for *HOXA1*, *ADGRF1* and *MATK*, all the genes of interest showed a reversed pattern when vimentin was downregulated in MCF-7FV. Blue: Positive log2-fold value, RED: Negative log2-fold value.

Gene	MCF-7FV (log2 fold)	MCF-7FV_ shVIM (log2 fold)	Fold Change	Expression Reversed
VIM	9.33650656	-1.986284197	11.32279076	Yes
C15orf59-AS1	4.786779989	0.649618272	4.137161717	Yes
RAMP3	1.25038923	0.096330197	1.154059033	Yes
ITGA2B	2.290069181	0.811826829	1.478242353	Yes
WISP2	3.433903727	-0.808460004	4.242363731	Yes
FGFR4	3.714285714	-0.241645848	3.955931562	Yes
LINC00052	2.979911014	-4.440209806	7.42012082	Yes
KIF26A	2.566210046	-2.199698346	4.765908392	Yes
NES	1.996379684	-1.262787239	3.259166923	Yes
CDC45	1.9806455	0.243201387	1.737444113	Yes
MATK	2.306631192	3.442590336	**-1.135959144**	**NO**
CDC20	1.587745325	-1.281117448	2.868862773	Yes
FOXM1	2.366990834	0.566254483	1.800736351	Yes
TUBG1	1.814550423	0.178771814	1.635778609	Yes
CDCA3	2.50370981	0.146757782	2.356952028	Yes
BCL2	1.356248168	-0.71699422	2.073242388	Yes
FSD1	1.177538186	-1.433959369	2.611497555	Yes
E2F1	0.854875084	-2.010915233	2.865790317	Yes
COL4A3	-1.745457055	-0.088918639	1.656538416	Yes
CDH10	-2.018109381	0.629736673	2.647846053	Yes
CD109	-3.707102794	1.02485821	4.731961004	Yes
TFPI2	-2.496937753	3.745604854	6.242542607	Yes
TGFBI	-0.250201591	0.383419968	0.633621559	Yes
CEACAM1	-7.111771511	3.514936088	10.6267076	Yes
PTPRM	-3.997693533	-2.95325812	1.044435413	Yes
AXL	-0.820199455	-0.116622441	0.703577014	Yes
CDH5	-4.912386583	2.088590387	7.000976971	Yes
ADGRF1	-3.41636036	-4.595092856	**-1.178732495**	**NO**
HOXA1	-0.813012192	-6.697688355	**-5.884676163**	**NO**

Table S12: Comparison of relative log2-Fold Changes in DEGs of interest between MCF-7FV and MDA-MB-231_shVIM. The relative log2-fold changes in DEGs between MCF-7FV and MDA-MB-231_shVIM was calculated to determine "reversibility" in their expression after vimentin knockdown by shVIM RNA. The gene expression was considered "reversed" if positive log2-fold values in MCF-7FV became negative or going towards negative in MDA-MB-231_shVIM and vice versa. Except for *CDC20*, all the genes of interest showed a reversed pattern when vimentin was downregulated in MDA-MB-231. Blue: Positive log2-fold value, RED: Negative log2-fold value.

Gene	MCF-7FV (log2 fold)	MDA-MB-231_shVIM (log2 fold)	Fold Change	Expression Reversed
VIM	9.33650656	-1.06806603	10.40457259	Yes
C15orf59-AS1	4.786779989	0.3382803	4.448499689	Yes
RAMP3	1.25038923	-1.574470127	2.824859357	Yes
ITGA2B	2.290069181	-0.704091069	2.99416025	Yes
WISP2	3.433903727	-0.483110208	3.917013936	Yes
FGFR4	3.714285714	0.155976368	3.558309346	Yes
LINC00052	2.979911014	-0.150223087	3.130134101	Yes
KIF26A	2.566210046	-0.71014298	3.276353026	Yes
NES	1.996379684	-1.303019768	3.299399452	Yes
CDC45	1.9806455	0.148930259	1.831715241	Yes
MATK	2.306631192	-1.224224881	3.530856073	Yes
CDC20	1.587745325	1.530771443	**0.056973881**	**No**
FOXM1	2.366990834	0.275577779	2.091413055	Yes
TUBG1	1.814550423	-0.436016392	2.250566815	Yes
CDCA3	2.50370981	-0.505576938	3.009286748	Yes
BCL2	1.356248168	-0.152490408	1.508738576	Yes
FSD1	1.177538186	-2.055766577	3.233304763	Yes
E2F1	0.854875084	-0.152490408	1.007365492	Yes
COL4A3	-1.745457055	0.101260246	1.846717301	Yes
CDH10	-2.018109381	0.245756414	2.263865795	Yes
CD109	-3.707102794	-1.758774742	1.948328052	Yes
TFPI2	-2.496937753	-0.267394408	2.229543345	Yes
TGFBI	-0.250201591	0.07412735	0.324328941	Yes
CEACAM1	-7.111771511	-1.695913218	5.415858293	Yes
PTPRM	-3.997693533	-0.555775161	3.441918372	Yes
AXL	-1.620199455	-0.695394998	0.924804457	Yes
CDH5	-4.912386583	0.103196916	5.015583499	Yes
ADGRF1	-3.41636036	1.209095599	4.625455959	Yes
HOXA1	-0.813012192	1.510372669	2.323384861	Yes

Table S13: Heatmap generated by comparison of relative log2-Fold Change of genes of interest in MCF-7FV_shVIM and MDA-MB-231_shVIM. Colour key: lowest= red, highest= yellow. DEGs that expressed similar log2-fold change (gene names in red colour) in both cell systems include, *CDH5*, *AXL*, *PTPRM*, *TGFBI*, *CDH10*, *E2F1*, *FSD1*, *BCL2*, *FOXM1*, *CDC45*, *NES*, *KIF26A*, *LIN00052*, *WISP2*, *C15ORF9-AS1* in addition to *VIM* gene implying a common gene signature regulated by vimentin.

Lowest **Highest**

	MCF-7FV_ shVIM (log2 fold change)	MDA-MB-231_shVIM (log2 fold change)	Same Expression
HOXA1	-6.697688355	1.510372669	NO
ADGRF1	-4.595092856	1.209095599	NO
CDH5	2.088590387	0.103196916	Yes
AXL	-0.116622441	-0.695394998	Yes
PTPRM	-2.95325812	-0.555775161	Yes
CEACAM1	3.514936088	-1.695913218	NO
TGFBI	0.383419968	0.07412735	Yes
TFPI2	3.745604854	-0.267394408	NO
CD109	1.02485821	-1.758774742	NO
CDH10	0.629736673	0.245756414	Yes
COL4A3	-0.088918639	0.101260246	NO
E2F1	-2.010915233	-0.152490408	Yes
FSD1	-1.433959369	-2.055766577	Yes
BCL2	-0.71699422	-0.152490408	Yes
CDCA3	0.146757782	-0.505576938	NO
TUBG1	0.178771814	-0.436016392	NO
FOXM1	0.566254483	0.275577779	Yes
CDC20	-1.281117448	1.300771443	NO
MATK	3.442590336	-1.224224881	NO
CDC45	0.243201387	0.148930259	Yes
NES	-1.262787239	-1.303019768	Yes
KIF26A	-2.199698346	-0.71014298	Yes
LINC00052	-4.440209806	-0.150223087	Yes
FGFR4	-0.241645848	0.155976368	NO
WISP2	-0.808460004	-0.483110208	Yes
ITGA2B	0.811826829	-0.704091069	NO
RAMP3	0.096330197	-1.574470127	NO
C15orf59-AS1	0.649618272	0.3382803	Yes
VIM	-1.986284197	-1.06806603	Yes
